# Genetic Models of Apoptosis-Induced Proliferation Decipher Activation of JNK and Identify a Requirement of EGFR Signaling for Tissue Regenerative Responses in *Drosophila*

**DOI:** 10.1371/journal.pgen.1004131

**Published:** 2014-01-30

**Authors:** Yun Fan, Shiuan Wang, Jacob Hernandez, Vildan Betul Yenigun, Gillian Hertlein, Caitlin E. Fogarty, Jillian L. Lindblad, Andreas Bergmann

**Affiliations:** 1 University of Massachusetts Medical School, Department of Cancer Biology, Worcester, Massachusetts, United States of America; 2 Graduate Program in Developmental Biology, Baylor College of Medicine, Houston, Texas, United States of America; 3 MD Anderson Cancer Center, Department of Biochemistry & Molecular Biology, Houston, Texas, United States of America; 4 Länderinstitut für Bienenkunde, Humboldt Universität zu Berlin, Hohen Neuendorf, Germany; Harvard Medical School, Howard Hughes Medical Institute, United States of America

## Abstract

Recent work in several model organisms has revealed that apoptotic cells are able to stimulate neighboring surviving cells to undergo additional proliferation, a phenomenon termed apoptosis-induced proliferation. This process depends critically on apoptotic caspases such as Dronc, the Caspase-9 ortholog in *Drosophila*, and may have important implications for tumorigenesis. While it is known that Dronc can induce the activity of Jun N-terminal kinase (JNK) for apoptosis-induced proliferation, the mechanistic details of this activation are largely unknown. It is also controversial if JNK activity occurs in dying or in surviving cells. Signaling molecules of the Wnt and BMP families have been implicated in apoptosis-induced proliferation, but it is unclear if they are the only ones. To address these questions, we have developed an efficient assay for screening and identification of genes that regulate or mediate apoptosis-induced proliferation. We have identified a subset of genes acting upstream of JNK activity including Rho1. We also demonstrate that JNK activation occurs both in apoptotic cells as well as in neighboring surviving cells. In a genetic screen, we identified signaling by the EGFR pathway as important for apoptosis-induced proliferation acting downstream of JNK signaling. These data underscore the importance of genetic screening and promise an improved understanding of the mechanisms of apoptosis-induced proliferation.

## Author Summary

Work in recent years has revealed that apoptotic caspases not only induce apoptosis, but also have non-apoptotic functions. One of these functions is apoptosis-induced proliferation, a relatively recently discovered phenomenon by which apoptotic cells induce proliferation of surviving neighboring cells. This phenomenon may have important implications for stem cell activity, tissue regeneration and tumorigenesis. Here, we describe the development of a genetic model of apoptosis-induced proliferation and the use of this model for convenient and unbiased genetic screening to identify genes involved in the process. We tested mutants of our RNAi transgenic lines targeting the core components of the apoptotic pathway and of JNK signaling, a known mediator of apoptosis-induced proliferation. These assays demonstrate the feasibility of the system for systematic genetic screening and identified several new genes upstream of JNK that are involved in apoptosis-induced proliferation. Finally, we tested the model in a pilot screen for chromosome arm 2L and identified *spi*, the EGF ligand in flies, as important for apoptosis-induced proliferation. We confirmed the involvement of EGF in a genuine apoptosis-induced regeneration system. These data underscore the importance of genetic screening and promise an improved understanding of the mechanisms of apoptosis-induced proliferation and regeneration.

## Introduction

Apoptosis is the major form of programmed cell death. It is used during development and under stress conditions to remove excess, unwanted or damaged cells. Deregulated apoptosis can give rise to malignancies including cancer and neurodegeneration [1]. A central step for the execution of apoptosis is the activation of caspases, a family of cysteine-proteases that are ubiquitously expressed as inactive zymogens [2]. There are two different types of caspases. Initiator caspases are activated by incorporation into multimeric complexes such as the apoptosome [3] in response to developmental signals, cellular stress and injury. The initiator caspase complex cleaves and activates effector caspases which then proteolytically process a large number of cellular proteins inducing the death of the cell.

Caspases are very well conserved in the animal kingdom. Of the seven caspases in *Drosophila*, only the initiator caspase Dronc and the two effector caspases DrICE and Dcp-1 have been implicated in apoptosis in imaginal discs [4]–[12]. Caspases are negatively regulated by inhibitor of apoptosis proteins (IAP) which directly bind to processed caspases and inhibit their activity [13]. *Drosophila* IAP1 (Diap1) binds to and inhibits Dronc, DrICE and Dcp-1 [14], [15]. In cells committed to die, IAP-antagonists such as Reaper, Hid and Grim [16]–[18] promote ubiquitin-mediated degradation of Diap1, thus releasing Dronc, DrICE and Dcp-1 from Diap1 inhibition [19]–[23]. Dronc associates with the scaffolding protein Ark (Apaf-1 related killer) to form the apoptosome which triggers activation of DrICE and Dcp-1.

Developing organisms have the ability to compensate for massive apoptotic cell loss by inducing compensatory proliferation. For example, developing *Drosophila* imaginal discs can form a normal-sized and patterned organ even after more than 50% of their cells have been killed by X-ray treatment due to compensatory proliferation [24]. Surprisingly, work in *Drosophila*, and later in hydra, Xenopus, planarians, newt and mice, has revealed that apoptotic caspases may be the driving force for compensatory proliferation in apoptotic tissue [12], [25]–[35] (reviewed in [36]–[38]). Because this regenerative proliferation requires apoptotic caspases, it has been termed Apoptosis-induced Proliferation, henceforth referred to as AiP [38], [39].

There are two commonly used experimental models that study AiP in larval imaginal discs, usually wing and eye discs in *Drosophila*. The first type of model takes advantage of the fact that another caspase inhibitor, the P35 protein from Baculovirus, specifically inhibits the effector caspases DrICE and Dcp-1, but not the initiator caspase Dronc [14], [40], [41]. Therefore, induction of apoptosis in *p35*-expressing cells triggers the apoptotic pathway down to Dronc, but cannot execute cell death because of inhibition of effector caspases by P35. These cells are referred to as ‘undead’ cells. Consequently, Dronc is functional in ‘undead’ tissues and can fulfill non-apoptotic roles including AiP which triggers overgrowth [12], [25]–[28] which may be relevant for tumorigenesis. More, recently, P35-independent models of AiP have been described [42]–[44]. In these models, apoptosis is temporally induced followed by analysis of the events leading to replacement of the dying tissue. Because they mimic the conditions of normal regenerative growth, we referred to them as ‘genuine’ AiP models [45].

Much of what we know about AiP came from studies of ‘undead’ cells. In ‘undead’ cells, Dronc activates *p53* and the stress-kinase JNK, encoded by *basket* (*bsk*) in *Drosophila* [12], [25]–[27], [46], [47]. JNK activity is both necessary and sufficient to induce AiP, and it may do this by expression of the Wnt family members *wingless* (*wg*) and the TGFβ/BMP-family member *decapentaplegic* (*dpp*), both of which are potent mitogens [26], [27], [42], [43], [48]–[51]. There are similarities and differences between the ‘undead’ and ‘genuine’ models. Both models involve JNK signaling, but the location of JNK activity appears to be different. While it is believed that under *p35*-expressing conditions, JNK activity occurs only in ‘undead’ cells [27], this is less clear in the ‘genuine’AiP model. Initially, it was reported that JNK is activated only in neighboring surviving cells [43]. More recently, it was shown that JNK is activated in both apoptotic and neighboring, surviving cells [44]. The role of Wg and Dpp in both models is also unclear. *wg* is not induced in all ‘undead’ cells and concern has been raised about the involvement of *wg* and *dpp* in ‘genuine’ AiP [26], [44], [46] suggesting that other signaling pathways are also critical for AiP.

There are many other open questions in the field. For example, although it is well established that Dronc can stimulate JNK activity, the molecular mechanism of this interaction is not known. Furthermore, while JNK is best characterized for its ability to induce apoptosis [52], it is not always known how JNK induces proliferation [50], [53]. For example, while in wing imaginal discs, JNK stimulates proliferation through activation of Yorkie, the downstream target of the Hippo growth control pathway, this does not appear to be a mechanism in eye imaginal discs [54], [55], the preferred model of this study (see below). This question is also relevant for understanding of tumorigenesis, as for example death receptor signaling by Fas (CD95) can promote tumor growth through JNK-induced proliferation [56]. These considerations stress the necessity of a convenient genetic screening system to identify the genes and mechanisms involved in AiP.

Here, we present and test the feasibility of ‘undead’ and ‘genuine’ genetic models of AiP in eye imaginal discs. We identify additional components in the JNK pathway that mediate the activation of JNK by Dronc. We show that JNK activation occurs in dying cells as well as in neighboring surviving cells depending on the conditions used. We report the results of a pilot screen using the ‘undead’ AiP model that led to the identification of Spi/EGFR signaling as essential component for AiP. Finally, we demonstrate that Spi is at least partially required for regeneration in a ‘genuine’ AiP model of the eye disc.

## Results

## The *ey>hid-p35* model

**Figure 1:**
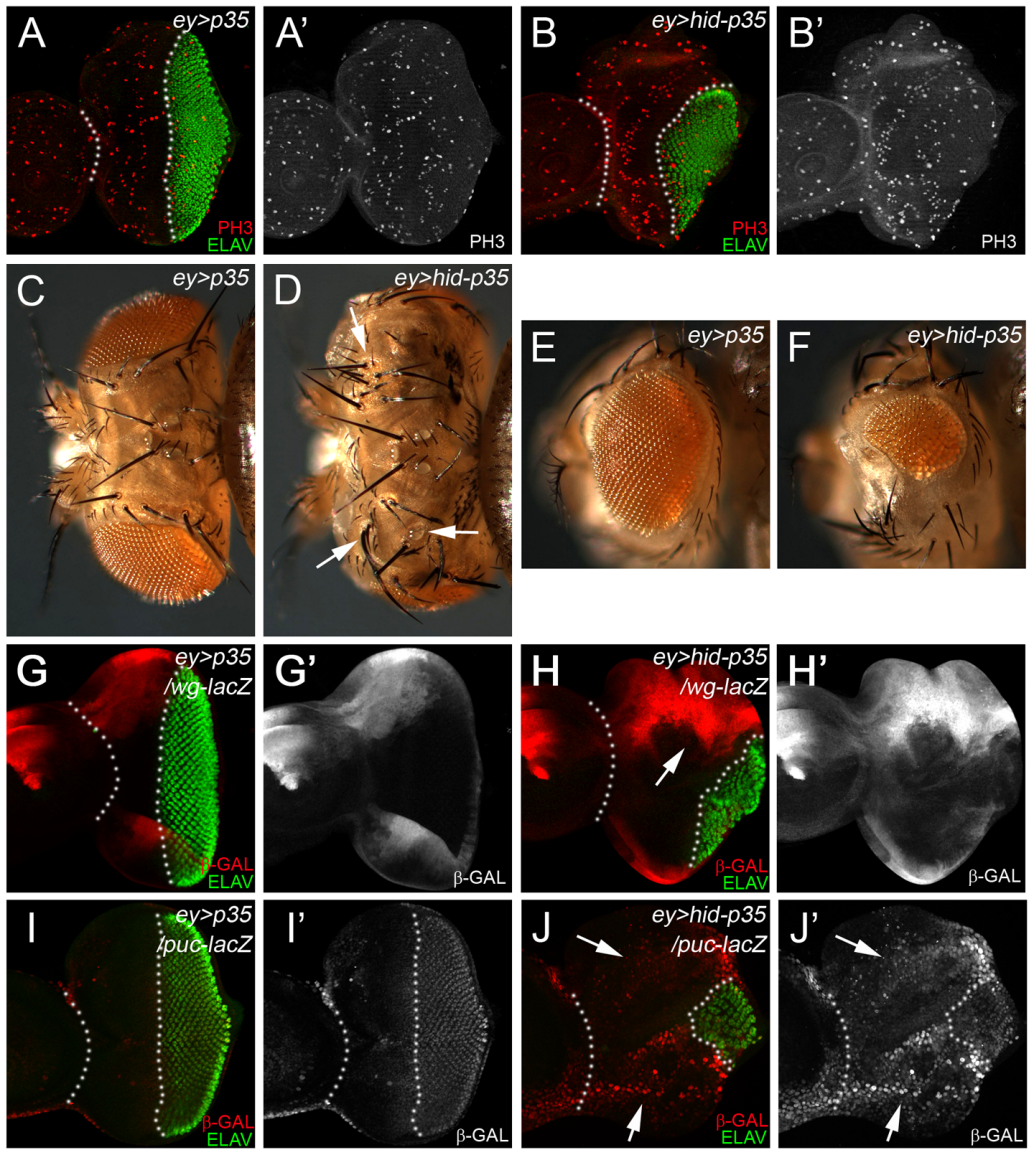
The *ey>hid-p35* model induces hyperplastic overgrowth and displays markers of apoptosis-induced proliferation. In this and the following figures, anterior is to the left. White dotted lines indicate the anterior portion of the eye imaginal discs. ELAV labels photoreceptor neurons and is used to mark the developing eye field posterior to the morphogenetic furrow (MF). (A,A′) An *ey>p35* control eye disc labeled with PH3 as proliferation marker (red in A; grey in A′) and ELAV (green in A). (B,B′) An *ey>hid-p35* experimental disc labeled with PH3 (red in B; grey in B′) and ELAV (green in B). Please note the increase in size of the region anterior to the MF at the expense of the posterior region (green). (C,D,E,F) Dorsal views of heads (C,D) and eyes (E,F) of *ey>p35* control (C,E) and *ey>hid-p35* experimental flies (D,F). Enlarged head cuticle with additional ocelli and bristles (arrows) is observed in *ey>hid-p35* flies (D), while eyes are reduced in size (F). (G,G′,H,H′) Increased expansion of *wg* expression (*wg*-*lacZ*, red in G,H; grey in G′,H′) in *ey>hid-p35* discs (H, arrow) compared to *ey>p35* control discs (G). (I,I′) In *ey>p35* control discs, *puc*-*lacZ* expression (β-Gal; red in I, gray in I′) as marker of Bsk/JNK activity is low anterior to the MF and induced posterior to the MF. (J,J′) *puc*-*lacZ* expression (β-Gal; red in J, gray in J′) as marker of Bsk/JNK activity is strongly induced anterior to the MF in *ey>hid-p35* eye discs (arrows). Note the reduction in the posterior eye field as visualized by ELAV labeling (green).

*eyeless* (*ey*) is a regulatory gene for eye development and is expressed during the growth phase of eye imaginal discs [57]. With the move of the morphogenetic furrow (MF) in 3^rd^ instar larvae, *ey* expression ceases in and posterior to the MF [57]. Therefore, co-expression of *hid* and *p35* during the growth phase of the eye disc using *ey-Gal4* (referred to as *ey>hid-p35*) may provide a convenient model to induce AiP. Correspondingly, in eye imaginal discs, the anterior portion of the eye disc is overgrown compared to controls forming an expanded head capsule due to increased cell proliferation [29] (Figure 1A,B,C,D). Additional ocelli and bristles are observed (Figure 1D, arrow). The anterior overgrowth is at the expense of posterior tissue (Figure 1A,B) which specifies the retina. As a result, eyes are smaller than wild-type and often absent (Figure 1E,F). In eye discs, we use ELAV labeling which labels photoreceptor neurons, as a marker to assess the extent of anterior overgrowth and distortion of the eye disc (Figure 1B,G). We refer to these phenotypes as AiP phenotypes. The small eye tissue is likely due to the expansion of Wg expression anterior to the MF (Figure 1G,H) which is an inhibitor of MF progression [58]. We also observed anterior expansion of *dpp-lacZ* expression [29]. Finally, expression of the JNK marker *puc-lacZ* and *TRE-dsRed* [59] are strongly expanded anterior to the MF (Figure 1I,J; Suppl. Figure S1). Therefore, known markers of AiP are induced in the *ey>hid-p35* model which therefore may represent a convenient AiP model for genetic screening.

### The *ey>hid-p35* model requires the apoptosome components *dronc* and *ark*, but is independent of effector caspases

**Figure 2:**
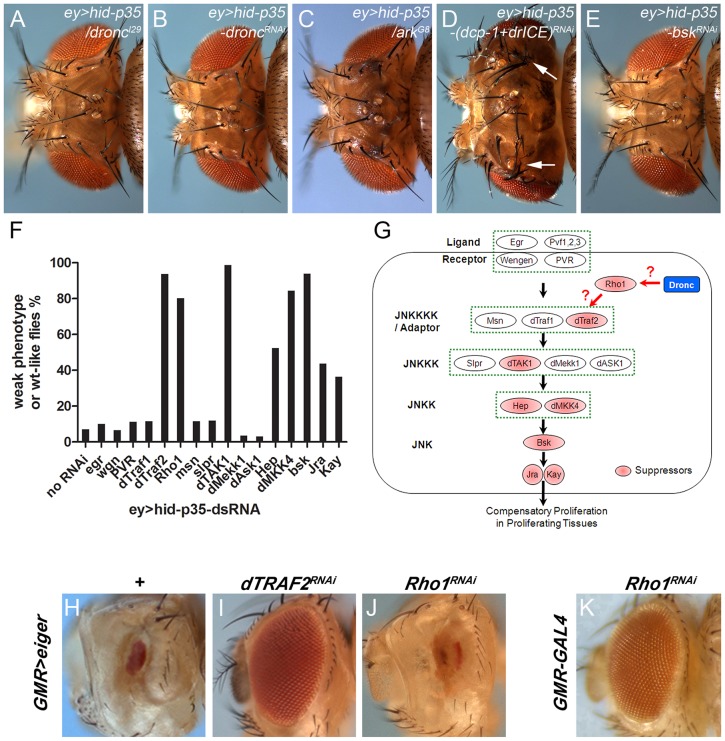
Modification of the *ey>hid-p35* phenotype by JNK pathway components. (A–E) *dronc* (A) and *ark* (C) heterozygosity strongly suppresses the *ey>hid-p35* phenotype (compare to Figure 1D). RNAi targeting *dronc* (B) and *bsk* (E) also strongly suppresses it. Double RNAi targeting *dcp-1* and *drICE* (D) has no effect. (F) Results of the suppression of *ey>hid-p35* using RNAi targeting components of the Bsk/JNK pathway in *Drosophila*. Only select members of the Bsk/JNK pathway (*dTraf2*, *Rho1*, *dTAK1*, *dMKK4*, *Bsk* and to a weaker extent *hep*, *Jra* and *kay*) show suppression. Each RNAi analysis was repeated at least twice with scoring more than 50 *ey*>*hid*-*p35*/*dsRNA* adult flies. (G) Schematic summary of the suppression analysis of the Bsk/JNK pathway. Pathway components highlighted in red show RNAi-mediated suppression and are thus required for *ey>hid-p35*-induced proliferation. (H–J) The *GMR*>*eiger*-induced eye ablation phenotype (H) is strongly suppressed by *dTRAF2* RNAi (I), but not by *Rho1* RNAi (J). (K) *GMR-Gal4* driven RNAi targeting *Rho1* does not cause an eye ablation phenotype. This control experiment shows that failure of *Rho1* RNAi to suppress *GMR*>*eiger* (J) is not due to a secondary effect.

To test the feasibility of the *ey>hid-p35* model for genetic screening, we first examined if mutants and RNA interference (RNAi) of caspases and *ark* genetically modify the AiP phenotype. Heterozygosity of *dronc* and *dronc* RNAi strongly suppressed the AiP phenotype (Figure 2A,B). Under these conditions, more than 95% of the flies display completely normal eye and head morphology. Heterozygosity of the apoptosome component *ark* also suppresses the AiP phenotype to a similar extend (Figure 2C). Therefore, these data demonstrate that the *ey>hid-p35* model is sensitive to genetic alterations and extend previous findings that AiP not only requires *dronc*, but also *ark*, i.e. a functional apoptosome.

Because this type of AiP is dependent on effector caspase inhibition by P35, it was inferred that it does not require effector caspases [25], [27], [28]. However, it was recently suggested that despite P35 inhibition, effector caspases may still be active at low levels in ‘undead’ cells [60]. This low level effector caspase activity may be insufficient to induce apoptosis, but sufficient to trigger non-apoptotic functions such as invasive behavior of ‘undead’ cells [60]. To test this possibility for AiP, we further reduced DrICE and Dcp-1 activity by double RNAi due to the redundancy of these two effector caspases [8]. However, in contrast to the invasive behavior of ‘undead’ cells [60], the AiP phenotype was not suppressed by *dcp-1;drICE* double RNAi (Figure 2D). The RNAi stocks used are functional as *dcp-1*;*drICE* double RNAi suppresses *hid* activity in a different apoptotic model, *GMR-hid* (Suppl. Figure S2). In summary, the overgrowth of the *ey>hid-p35* model is dependent on the apoptosome components Dronc and Ark, but independent of effector caspases.

### Identification of JNK pathway components involved in AiP

It is unknown how the apoptosome induces JNK activity for AiP. To obtain further insight into this question, we tested components of the JNK pathway in a pilot RNAi screen for modification of the *ey>hid-p35* model. As expected, RNAi targeting *bsk*, the JNK ortholog in *Drosophila*, completely suppresses the AiP phenotypes in more than 90% of the flies (Figure 2E,F,G). Downstream of JNK, RNAi knockdown of the components of the AP1 transcription factor, *jun-related antigen* (*jra*) and the Fos ortholog *kayak* (*kay*), also suppressed the *ey>hid-p35* AiP phenotypes, although to a lesser extent (Figure 2F,G) suggesting that they are at least partially required for AiP.

To identify upstream components in the JNK pathway involved in AiP, we tested RNAi lines targeting all known components in the JNK pathway [52]. Interestingly, only a subset of them were found to suppress the AiP phenotypes (Figure 2F,G). This includes the JNKKK *dTak1* and the JNKKs *hemipterous* (*hep*) and *MKK4* (Figure 2F,G). The non-redundant functions of *hep* and *MKK4* for AiP is puzzling, but has been previously reported in different contexts [61], [62]. Further upstream in the JNK signaling pathway, we only identified *Traf2* (also known as *Traf6*) as AiP suppressor (Figure 2F,G). Another regulator of JNK signaling, the small GTPase *Rho1* [63]–[66], was also identified as AiP suppressor. In contrast, the two ligand/receptor systems known to activate JNK, Eiger/Wengen and Pvf/PVR, do not suppress AiP (Figure 2F,G). The RNAi lines against these genes are functional as shown in Suppl. Figure S3 and in [67]–[69].

Theoretically, it is possible that the suppression of AiP by these RNAi transgenes is an indirect result of suppression of apoptosis, as observed in the case of *dronc* and *ark* mutants or RNAi (Figure 2B,C; Suppl. Figure S4B). To exclude this possibility, we labeled *ey*>*hid*-*p35* eye imaginal discs expressing these RNAi constructs with cleaved Caspase-3 (Cas3*) antibody, a marker of Dronc activity [70], and ELAV antibody to evaluate rescue of disc morphology. Despite the rescue of disc morphology, Cas3* labeling is not significantly suppressed by these RNAi constructs (Suppl. Figure S4C–H) suggesting that the suppression of the AiP phenotype by reducing JNK activity is not due to suppression of caspase activity.

Because *Rho1* is the least well characterized regulatory component in the JNK pathway, we examined the effect of *Rho1* knockdown on JNK activity in the AiP model. Loss of *Rho1* suppresses *puc*-*lacZ* in *ey*>*hid*-*p35* eye discs (Suppl. Figure S5A,B). *Rho1* RNAi also suppresses the AiP marker Wg (Suppl. Figure S5C,D). These data show that *Rho1* acts genetically upstream of JNK in the AiP model consistent with previous reports [63]–[66].

To further place *Rho1* into the AiP pathway and to relate it to *Traf2*, we examined the ability of *Rho1* and *Traf2* to suppress *GMR*-*eiger*, a known inducer of JNK activity causing a strong eye ablation phenotype (Figure 2H) [71], [72]. Interestingly, while *Traf2* knockdown effectively suppresses *GMR-eiger* as reported [73], *Rho1* RNAi does not (Figure 2I,J). It is possible that *Rho1* RNAi disrupts eye development by itself and that may be the reason for the failure to suppress *GMR-eiger*. However, *Rho1* RNAi does not disrupt eye development (Figure 2K). These observations suggest that the role of Rho1 for JNK activation is independent of Eiger which is also consistent with the observation that *Eiger* knockdown does not suppress AiP (Figure 2F,G). Furthermore, these data raise the possibility that Traf2 serves as an integration point of both Eiger signaling and AiP for JNK activation. For these reasons, we place *Rho1* upstream of *Traf2* in the AiP pathway (Figure 2G), but there may also be other ways by which Rho1 controls JNK activation.

### Activation of JNK signaling in ‘undead’/dying cells and neighboring, surviving cells

The lack of a requirement of Eiger/Wengen and Pvf/PVR in our AiP model (Figure 2F,G) may suggest that activation of JNK occurs in dying cells. However, conflicting data have been reported about the location of JNK activity in various AiP models. Initially, JNK signaling was observed in ‘undead’ cells [27]. In a *p35*-independent regeneration model, it was reported that JNK signaling occurs only in neighboring surviving cells [43]. More recently, JNK activity was reported to be both in dying and neighboring surviving cells [44]. While there are experimental differences between these studies, none of them used a mosaic approach to determine the location of JNK activation. Therefore, to clarify this issue, we re-examined both ‘undead’ and ‘genuine’ AiP models for location of JNK activity.

**Figure 3:**
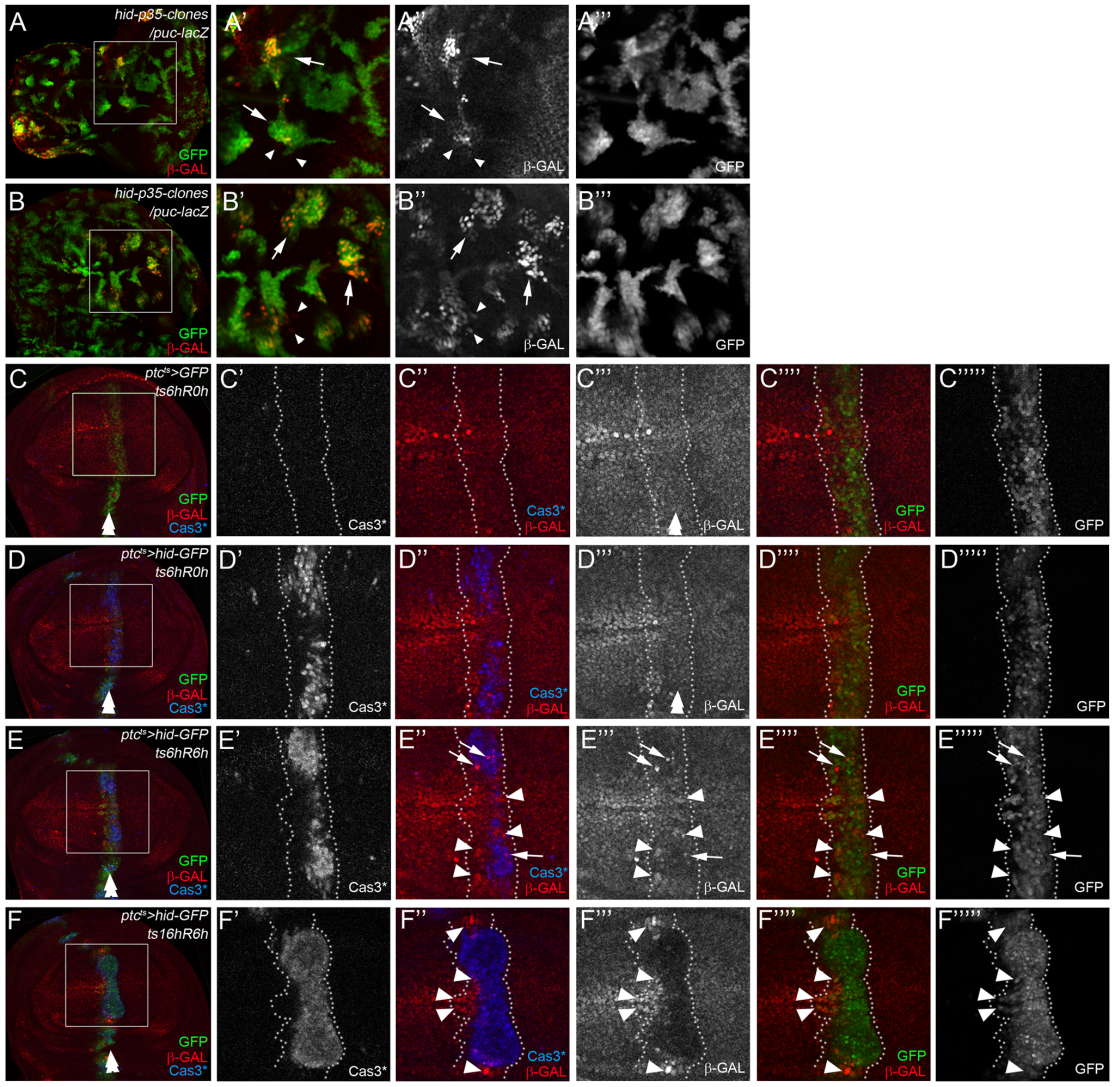
Location of Bsk/JNK signaling in ‘undead’ and ‘genuine’ AiP models. *puc*-*lacZ* was used as JNK activity marker. *hid* expressing areas are marked by GFP. Arrows indicate JNK activity in ‘undead’/dying cells, while arrowheads mark JNK activity in surviving cells. A double arrow in (C–F) marks the *ptc* domain. (A,B) Location of JNK signaling in ‘undead’ AiP models by mosaic analysis. Clones expressing *hid* and *p35* were induced by FLP-out technology. In eye (A) and wing (B) imaginal discs, *puc-lacZ* expression is mostly induced in *hid*/*p35*-expressing clones (arrows in A′, A″,A′″, B′, B″,B′″). However, a few examples of *puc*-*lacZ* expression are noted in cells outside of *hid*/*p35*-expressing clones (arrowheads). (C–F) Location of JNK activity in a ‘genuine’ AiP model in wing imaginal discs. *hid* expression was under control of *ptc-Gal4* and *tub*-*Gal80^ts^* (*ptc^ts^*>*hid*). A temperature shift (ts) to 30°C for the indicated amount of time during 3^rd^ larval instar induced *hid* expression. After the indicated recovery period (R), discs were labeled for GFP (to visualize the *ptc* domain), Cas3* (the death domain) and β-Gal (*puc*-*lacZ*, i.e. JNK activation). The *ptc* domain is outlined by white, dotted lines. Note that the death domain does not completely overlap with the *ptc* domain (see for example E′,E′″″ and F′,F′″″). (C–C′″″) A control disc just expressing *GFP* under the experimental conditions to visualize the normal *puc*-*lacZ* pattern (β-Gal) pattern. (D–D′″″) An experimental disc that was dissected immediately after a 6 hours pulse of *hid* expression without recovery (ts6hR0h). While caspase activity has been induced (D′), the *puc*-*lacZ* pattern is mostly unaffected (D′″). (E–E′″″) An experimental disc that was allowed to recover for 6 hours after a 6 hours pulse of *hid* expression (ts6hR6h). *puc*-*lacZ* (β-Gal) expression is induced in many cells both inside (arrows) and outside (arrowheads) of the death domain (E″,E′″). However, these cells are present in the *ptc* domain (GFP; E″″,E′″″). (F–F′″″) An experimental disc that was allowed to recover for 6 hours after a pulse of *hid* expression for 16 hours (ts16hR6h). Although apoptosis is now strongly induced (F′), it is not detectable in the entire *ptc* domain (GFP; F′″″) suggesting that some cells can escape *hid*-induced cell death. *puc*-*lacZ* (β-Gal) is strongly reduced in dying cells (F′″). Nevertheless, there is an increase of *puc*-*lacZ* expression in cells outside of the death domain (F″; arrowheads). However, these cells reside in the *ptc* expression domain (F″″,F′″″). Genotypes: (A,B) *hs-FLP*/*UAS-hid*; *UAS-p35*/*act*>*y^+^*>*Gal4 UAS-GFP*; *puc-lacZ*/+. (C) *ptc*-*Gal4 tub*-*Gal80^ts^*/+; *UAS*-*GFP*/*puc*-*lacZ*. (D–F) *UAS*-*hid*/+ ; *ptc*-*Gal4 tub*-*Gal80^ts^*/+; *UAS*-*GFP*/*puc*-*lacZ*.

In a mosaic ‘undead’ model, we expressed *hid* and *p35* in clones in eye and wing discs using a *FLP*-out approach and analyzed *puc-lacZ* expression as JNK reporter. GFP was used to mark *hid/p35*-expressing clones. Using this approach, *puc-lacZ* is predominantly expressed in *hid*/*p35* expressing cells (Figure 3A,B; arrows). However, we also noted a few examples where *puc-lacZ* was expressed in *GFP* ^−^ tissue (Figure 3A,B; arrowheads). These observations suggest that JNK activation occurs largely in ‘undead’ cells, but also in neighboring, normal cells.

To address this question in a ‘genuine’ (*p35*-independent) AiP model, we repeated the experiments by Bergantinos et al. (2010) [43] in wing imaginal discs. These authors reported JNK activity in neighboring surviving cells only. We induced *hid* in a temporally and spatially controlled manner using *ptc*-*Gal4* and *tub*-*Gal80^ts^* (*ptc^ts^*>*hid*) by temperature shifts for various times. In control experiments, just expressing *GFP* in the *ptc* domain does not affect the *puc-lacZ* pattern (Figure 3C,C′″). However, when *hid* expression was induced, depending on the conditions, different results were obtained regarding the location of JNK activity. In response to a short pulse (6 hours) of *hid* expression followed by a 6 hours recovery period (ts6hR6h), an elevation of *puc-lacZ* activity was detected in dying cells and neighboring, surviving cells (Figure 3E″, E′″; dying cells containing *puc-lacZ* are highlighted by arrows, while surviving cells are marked by arrowheads). This JNK activity was induced during the recovery period, because immediately after *hid* induction (ts6hR0h), no alteration of *puc-lacZ* expression was detected (Figure 3D′″). However, when *hid* expression was induced for a long period (16 hours) followed by 6 hours recovery (ts16hRh), *puc-lacZ* was strongly down-regulated in dying cells, likely as a result of apoptosis in these cells. Nevertheless, upregulation of *puc-lacZ* was detected in neighboring surviving cells (Figure 3F″, arrowheads). This result is consistent with Bergantinos et al. (2010) [43]. However, the upregulation of *puc-lacZ* still occurred in *GFP* ^+^ cells (Figure 3F″″,F′″″), i.e. in the *ptc* domain which had been exposed to *hid* 6 hours earlier, but have survived for unknown reasons. Similar results were observed in Figure 3E: the surviving cells inducing *puc-lacZ* are located in the *GFP* ^+^ region, i.e. in the *ptc* domain (Figure 3E″″,E′″″; arrowheads). Thus, it is not clear whether JNK activity in surviving cells is induced autonomously in response to *hid* expression, or by a signaling event from the dying Cas3*-positive cells. In any case, these data show that both in ‘undead’ and ‘genuine’ AiP models, JNK activity can be detected in ‘undead’/dying cells as well as in neighboring, surviving cells.

### Identification of *spi* as AiP suppressor

A systematic mutagenesis screen for genes involved in AiP has not been performed to date due to absence of a convenient screening assay. However, the data presented in Figure 2 demonstrate that suppression of *ey>hid-p35*-induced AiP provides a convenient assay for genetic screening. Therefore, as proof of principle, we screened a total of 106 chromosomal deficiencies deleting segments on the left arm of chromosome 2 (2L) for modification of the AiP phenotype and identified four chromosomal segments as dominant AiP suppressors and seven deficiencies as dominant AiP enhancers (Table 1; Suppl. Table S1), validating the deficiency approach. Enhancers display an even stronger AiP phenotype with severely overgrown head cuticle and strong semi-lethality.

**Table 1:** Deficiencies that modify the *ey>hid-p35*-induced AiP phenotype as suppressors or enhancers.

Suppressors of *ey*>*hid*-*p35*- induced Overgrowth	Chromosomal Location	Enhancers of *ey*>*hid*-*p35*-induced Overgrowth	Chromosomal Location
Df(2L)C144, Df(2L)ED136	22F4-23A2	Df(2L)ED123	22D1-22D3
Df(2L)ED206, Df(2L)JS17	23C4-23C5	Df(2L)BSC6	26D3-26E1
Df(2L)Exel7014, Df(2L)BSC28	23C4-23C5	Df(2L)BSC6	26D3-26E1
Df(2L)BSC31	23E5-23F3	Df(2L)ED508	28C1-28C4
Df(2L)TW137	?	Df(2L)ED611	29B4-29C3
Df(2L)ED1303, Df(2L)ED1272	37F2-38A2	Df(2L)Exel7048	31E3-31F4
		Df(2L)ED1050	35C1-35D1
		Df(2L)Exel7080	38F5-39A2

The indicated chromosomal location is the smallest overlap of overlapping deficiencies. *Df(2L)TW137* is marked with a “?” because other overlapping deficiencies do not suppress AiP (see Suppl. Table S1) indicating that the *Df(2L)TW137* chromosome carries a suppressor mutation independent of the deficiency.

**Figure 4:**
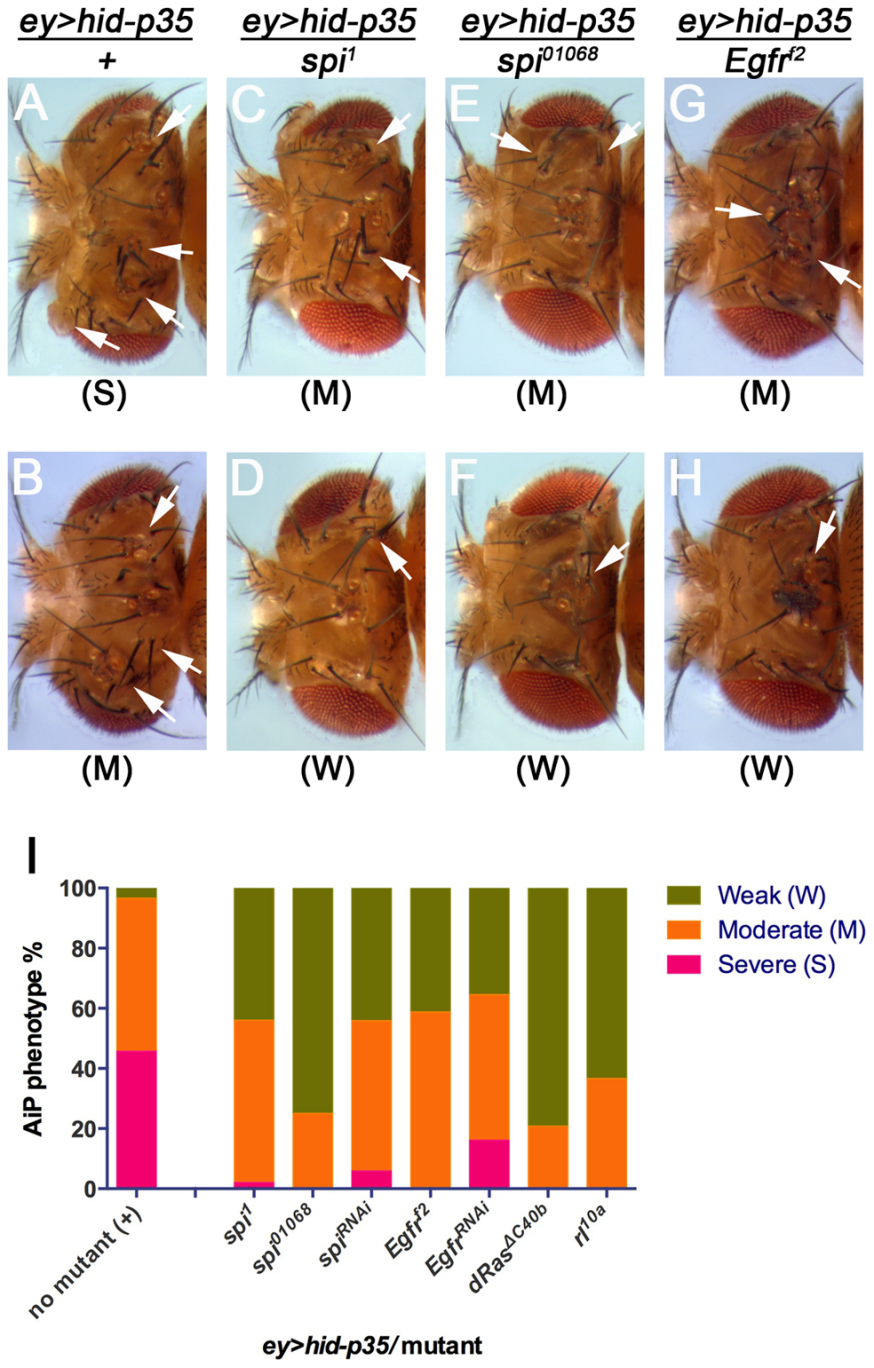
Suppression of *ey>hid-p35* by *spi* and *Egfr* inactivation. The hyperplastic phenotype of *ey>hid-p35* flies can be grouped in three categories, severe, moderate and weak. Flies were scored as severe when the head cuticle was strongly overgrown without discernible patterning and eyes were absent or strongly reduced in size. A moderate phenotype was scored when the head cuticle was overgrown, but recognizably patterned with duplicated ocelli and bristles. A weak phenotype was scored when size of head cuticle and eyes was almost normal with very few ectopic ocelli or bristles occasionally observed. (A–H) Representative pictures of *ey>hid-p35* fly head cuticles scored in different categories. Completely suppressed *ey>hid-p35* phenotype (wild-type-like head cuticles) by *spi* or *Egfr* heterozygotes are not shown here. Arrows indicate ectopic ocelli or bristles. (A,B) About 50% of *ey>hid-p35* flies show severe hyperplastic overgrowth of the head cuticle (A), while the remaining 50% display a moderate phenotype (B). (C–H) Heterozygosity of *spi^1^*, *spi^01068^* and *Egfr^f2^* almost completely eliminated the severe overgrowth phenotype of *ey>hid-p35* flies and largely extends the population of flies with a weak phenotype. (I) Summary of the suppression of the *ey>hid-p35* overgrowth phenotype in *spi*, *egfr*, *dRas* and *rolled (rl)* heterozygous condition. Pink indicates severe, orange indicates moderate and green indicates weak phenotypes. Mutant alleles are indicated.

To identify the genes in the deficiencies that dominantly cause the suppression of AiP, we tested available mutants and UAS-RNAi stocks against all genes that map to these deficiencies. This approach has been completed for *Df(2L)ED1303* (Table 1) and led to the identification of *spitz* (*spi*) as a potential regulator of AiP (compare Figure 4C–F,I with Figure 4A,B). *spi* encodes the EGF ortholog in *Drosophila* [74]. Therefore, our deficiency screen raises the hypothesis that the EGFR pathway regulates AiP. Consistently, heterozygosity of *Egfr* suppresses the *ey>hid-p35* phenotype in eye discs (compare Figure 4G–I with Figure 4A,B). We also found that *Egfr* RNAi suppresses an AiP model in wing imaginal discs (*nub*>*hid*-*p35*) (Suppl. Figure S6). Downstream of EGFR, mutant alleles of the *Drosophila* orthologs of Ras (*Dras*) and MAPK (*rolled* (*rl*)) act as dominant suppressors of *ey>hid-p35* (Figure 4I) suggesting that MAPK activity is required for AiP. These data imply that EGFR/Ras/MAPK signaling is essential for AiP in both eye and wing discs. These findings are exciting giving the controversy of the role of Wg and Dpp for AiP (see Introduction) [26], [46].

**Figure 5:**
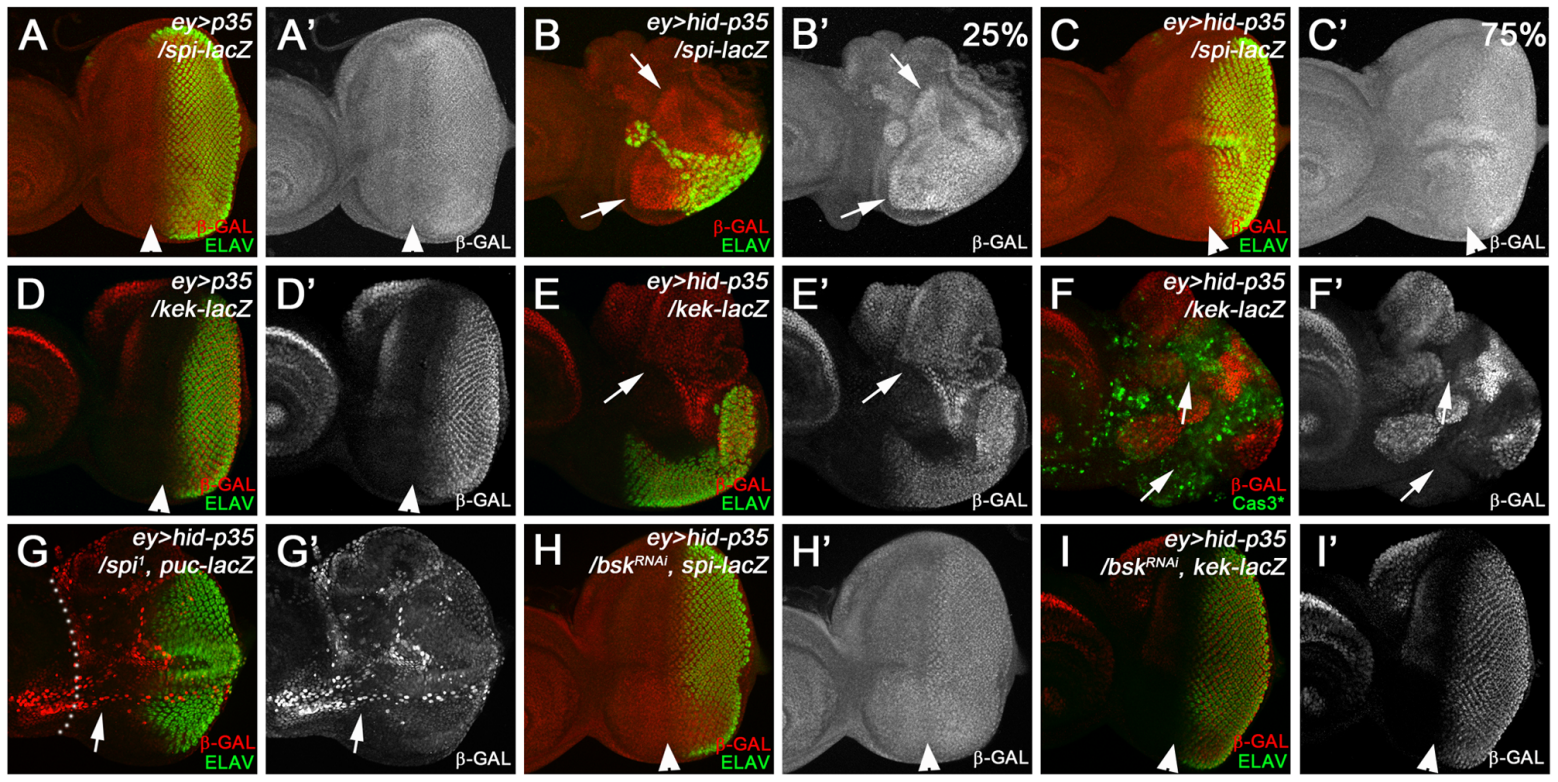
Epistasis analysis of *spi* and *bsk*. Arrowheads indicate the morphogenetic furrow (MF) which separates the anterior (left) from the posterior eye tissue visualized by ELAV labeling. (A) *spi-lacZ* pattern (β-Gal; red in A; gray in A′) in *ey>p35* control discs. Note there is little expression anterior to the MF. (B,C) Because the *spi-lacZ* allele (*spi^01068^*) is a suppressor of *ey>hid-p35* adult overgrowth phenotype (Figure 4I), there is variation in the β-Gal pattern. About 25% of the eye discs show strong induction of *spi*-*lacZ* in the anterior portion of the eye disc (B,B′; arrows) with a strong reduction of the eye field (ELAV). The remaining 75% of the eye discs show a suppressed, largely normal β-Gal and ELAV pattern in *ey>hid-p35* larvae (C,C′). This ratio corresponds to the suppression of the adult overgrowth phenotype (Figure 4I). (D,E) Strong induction of *kek*-*lacZ* (β-Gal; red in D,E; gray in D′,E′) in *ey>hid-p35* eye discs (E; arrows) compared to *ey>p35* control discs (D). (F,F′) *kek*-*lacZ* (β-Gal; red in F, gray in F′) is preferentially induced in patches of tissue adjacent to areas with high levels of active caspases (arrows, Cas3* in green). (G,G′) Heterozygosity of *spi* normalizes the eye field (ELAV, green), but does not suppress ectopic *puc*-*lacZ* expression (β-Gal; red in G, gray in G′) in *ey>hid-p35* eye discs (arrows, compare to Figure 1J). Dotted white lines outline the region anterior to the MF. (H,H′) Expression of *bsk^RNAi^* in *ey>hid-p35* discs normalizes the eye field (ELAV, green) and suppresses ectopic increase of *spi*-*lacZ* expression (β-Gal; red in H, gray in H′). This pattern was observed in all experimental discs (n = 30). (I,I′) Expression of *bsk^RNAi^* in *ey>hid-p35* discs normalizes the eye field (ELAV, green) and suppresses ectopic *kek*-*lacZ* expression (β-Gal; red in I, gray in I′; compare to E). The analysis in G, H and I strongly suggests that *spi* acts genetically downstream of *bsk*.

To further characterize the involvement of Spi/EGFR signaling for AiP, we took advantage of the *spi^01068^* allele which is an enhancer trap insertion of *lacZ* into the *spi* gene (*spi*-*lacZ*) and can serve as a reporter for *spi* expression [75]. This analysis is complicated by the fact that this *spi* allele itself is a dominant suppressor of AiP: about 75% of the *ey*>*hid*-*p35*/*spi^01068^* flies show a weak AiP phenotype, while 25% are not suppressed and still show a moderately strong AiP phenotype (Figure 4E,F,I). Consistently, in about 25% of *ey>hid-p35* eye discs (n = 28), we observed a strong induction of β-Gal labeling compared to control discs (Figure 5A,B). This percentage corresponds to the number of heterozygous *spi^01068^* flies which display a moderately strong AiP phenotype (Figure 4I). The remaining 75% of *ey*>*hid*-*p35* eye discs heterozygous for *spi^01068^* show a normalization of disc morphology as visualized by ELAV labeling and *spi-lacZ* expression (Figure 5C). In addition, we found that a target gene of the EGFR pathway, *kekkon-1(kek)* [76] is induced during AiP (Figure 5D,E,F).

To determine the position of Spi/EGFR signaling in the AiP pathway, we performed epistasis experiments between *spi* and *bsk*. Heterozygosity of *spi^1^* dominantly suppresses the adult AiP phenotype of *ey>hid-p35* (Figure 4C,D,I). This suppression can also be visualized by the normalization of the ELAV pattern in *ey>hid-p35* eye imaginal discs (Figure 5G, compare to Figure 1J). However, despite the normalization of the ELAV pattern, *puc-lacZ* expression is not reduced in this genetic background (Figure 5G,G′) suggesting that *spi* acts genetically downstream of *bsk*. This is further confirmed by the reciprocal experiment in which *bsk* RNAi completely normalizes the *spi-lacZ* pattern in *ey>hid-p35* background (Figure 5H). *bsk* RNAi also normalizes the *kek-lacZ* pattern in *ey>hid-p35* background (Figure 5I). These observations suggest that Spi/EGFR signaling acts genetically downstream of *bsk* activity. Because Spi is a secreted signaling molecule, these findings may imply that EGFR activation occurs in cells adjacent to apoptotic, JNK-activating cells. This assumption is directly confirmed by the observation that *kek-lacZ* activity, a downstream marker of EGFR signaling, and Cas3* labeling as apoptotic marker do not overlap (Figure 5F, arrows). In summary, these data imply that *spi* expression occurs downstream of Bsk/JNK activity and that EGFR signaling acts in signal-receiving, proliferating cells.

### Characterization of ‘genuine’ AiP in the eye imaginal disc: the *DE^ts^*>*hid* model

**Figure 6:**
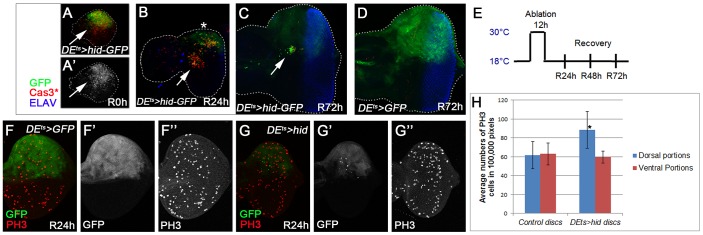
Characterization of ‘genuine’ AiP in the eye imaginal disc: the *DE^ts^*>*hid* model. *hid* expression was under control of *dorsal eye- (DE-)Gal4* and *tub*-*Gal80^ts^* (*DE^ts^*>*hid*). A temperature shift (ts) to 30°C for 12 h during 2^nd^ larval stage induced *hid* expression (E). After the indicated recovery period (R), discs were labeled for GFP (to visualize the *DE* expression domain), Cas3* (the death domain) and ELAV (to outline the shape of the disc). (A–C) *DE^ts^*>*hid* experimental discs. *hid* expression induces a strong apoptotic response (A) causing strong tissue loss after 24 h recovery in some discs (panel B; R24 h, asterisk). After 72 h recovery (R72 h), the disc has fully recovered and has a normal photoreceptor pattern as judged by ELAV labeling (C). Please note the strong reduction of GFP intensity which suggests that most of the *GFP* ^+^ cells have been replaced by new *GFP* ^−^ cells. Arrows highlight a patch of cells that are moving to the center of the disc. (D) A control disc 72 h after *DE^ts^*-induced GFP expression. Please note that GFP is a very stable protein that can still be detected 72 h after synthesis. (E) The protocol of the *DE^ts^*>*hid*-induced tissue ablation followed by recovery periods. (F,F′,F″,G,G′,G″) PH3-labeling of control (*DE^ts^*>*GFP*; F,F″) and experimental discs (*DE^ts^*>*hid*; G,G″). GFP marks the outline of the DE domain (F′,G′). (H) Quantification of the number of PH3-positive cells in dorsal and ventral compartments of control (F) and experimental discs (G). n = 40 for each genotype.

Finally, we tested if genes identified in the ‘undead’ (P35-dependent) AiP model are also involved in ‘genuine’ (P35-independent) regeneration in the eye disc. To accomplish this we used a similar approach as previously described in wing discs [42]–[44]. *hid* expression was spatially restricted to the dorsal half of the eye disc by *dorsal eye-Gal4* (*DE-Gal4*) [77] and controlled by *Gal80^ts^* [78] by a transient temperature shift (ts) to 30°C for 12 hours (Figure 6E). We refer to this system as *DE^ts^*>*hid*. This model also induces *GFP* to label *hid*-expressing cells. Before and after the temperature shift, animals were incubated at 18°C (Figure 6E) to inhibit Gal4 activity and therefore *hid* and *GFP* expression. Note that although *GFP* is expressed only during the 30°C pulse, it is a rather stable protein and can be detected in control discs 72 h later (Figure 6D).

In experimental discs immediately after the 30°C pulse (recovery 0 hours – R0 h), a strong apoptotic response is detectable (Figure 6A′) which causes tissue loss and disruption of the bilateral symmetry of the disc 24 hours later (R24 h). In extreme cases, this treatment can result in ablation of the entire dorsal half (Figure 6B, asterisk), but usually some dorsal tissue remains. At that time, many cells are still Cas3*-positive (Figure 6B). 72 hours after the temperature shift (R72 h), the disc has fully recovered in shape and also has a normal photoreceptor pattern as judged by ELAV labeling (Figure 6C). Cas3* activity is no longer detectable. The recovery is the result of increased proliferation in the dorsal half of the eye disc (compare Figure 6G″ to Figure 6F″; quantified in Figure 6H). The reduction of the GFP signal in the dorsal part (Figure 6C,G′) compared to the control disc (Figure 6D) suggests that most of the *GFP* ^+^ cells have died and have been replaced by new, *GFP* ^−^, cells.

Interestingly, a group of apoptotic cells appears to migrate out of the dorsal half into the center of the disc (Figure 6B; arrow). At R72 h, only these cells still show strong *GFP* ^+^-labeling (Figure 6C; arrow). This ‘escape’ response of these ‘genuine’ apoptotic cells is reminiscent of the invasive behavior of ‘undead’ cells in wing discs which move out of the ‘undead’ domain [60]. What makes these cells move is unknown, but an interesting avenue for further research in the future.

### Requirement of *bsk* and *spi* for regeneration in the ‘genuine’ AiP model *DE^ts^*>*hid*

Because JNK activity is essential for ‘undead’ AiP (Figure 2E), we examined a requirement of *bsk* in the ‘genuine’ (P35-independent) *DE^ts^*>*hid* model. First, we examined if JNK activity is induced in the *DE^ts^*>*hid* model. Consistent with the ‘genuine’ AiP models in the wing [42]–[44], this was indeed observed. *TRE-dsRed* as marker of JNK activity [59] peaked at 6 h after recovery (R6 h) and is still detectable at R12 h (Suppl. Figure S7B,C). It is mostly gone after 24 h recovery (Suppl. Figure S7D). *TRE-dsRed* is confined to the *GFP* ^+^ area, i.e. in the death domain (Suppl. Figure S7B,C).

**Figure 7:**
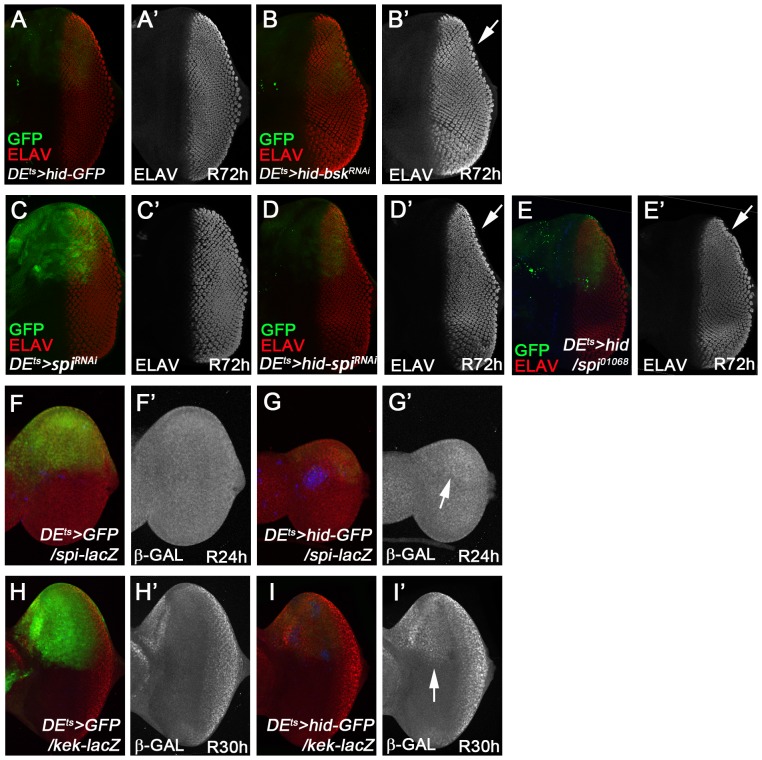
Requirement of *bsk* and *spi* for complete regeneration in the ‘genuine’ AiP model *DE^ts^*>*hid*. (A, A′) *DE^ts^*>*hid* discs treated following the protocol in Figure 6E fully recover after 72 h (R72H). n = 30. (A′) shows the ELAV-only channel. (B, B′) About 35% of *DE^ts^*>*hid* discs expressing *UAS*-*bsk* RNAi do not completely recover after 72 h. n = 25. The arrow in (B′) highlights the incomplete ELAV pattern on the dorsal half of the disc indicating that the regeneration response was partially impaired by reduction of *bsk* activity. Please note that this disc has also been labeled for GFP. (C, C′) A control eye disc expressing *UAS*-*spi* RNAi under *DE^ts^*-control following the protocol in Figure 6E. After 72 h recovery, the obtained ELAV pattern in the dorsal half of the eye disc is largely normal (red in C, gray in C′). n = 20. (D, D′) An experimental *DE^ts^*>*hid* eye disc that was simultaneously treated with *spi* RNAi. The arrow in (D′) highlights the incomplete ELAV pattern on the dorsal half of the disc indicating that the regeneration response was partially impaired by reduction of *spi* activity. 30 out of 30 discs show incomplete regeneration. Please note that this disc has also been labeled for GFP. (E, E′) An experimental *DE^ts^*>*hid* eye disc that was heterozygous for *spi^01068^*. Similar to (D), the ELAV pattern is incomplete on the dorsal half of the disc (E′, arrow). n = 20. (F, F′, G, G′) *spi-lacZ* pattern in control (*DE^ts^*>*GFP*; red in F, grey in F′) and experimental discs (*DE^ts^*>*hid*; red in G, grey in G′) at 24 h after recovery. The arrow in (G′) points to the increased β-Gal pattern in the dorsal half of the disc. Blue is Cas3*. (H, H′, I, I′) *kek-lacZ* pattern in control (*DE^ts^*>*GFP*; red in H, grey in H′) and experimental discs (*DE^ts^*>*hid*; red in I, grey in I′) at 30 h recovery. The arrow in (I′) points to the increased β-Gal pattern in the dorsal half of the disc. Blue is Cas3*.

Second, we determined if *bsk* is genetically required for tissue regeneration in the *DE^ts^*>*hid* model. We used the photoreceptor pattern (ELAV) as a marker to reveal disc outline and thus assess the degree of regeneration. Control discs (*DE^ts^*>*hid*) at R72 h had completely regenerated (Figure 7A; n = 30). However, if *bsk* was inactivated by RNAi during the apoptosis-inducing phase (Figure 6E), about 35% (9 of 25 discs) of the discs show incomplete regeneration (Figure 7B). The incomplete regeneration after *bsk* RNAi is weak, presumably because the 12 h down-regulation of *bsk* during the temperature shift is not sufficient to completely remove Bsk activity. It is also possible that Bsk is resynthesized quickly during the recovery period. Nevertheless, the incomplete regeneration after *bsk* RNAi suggests that Bsk is at least partially required for tissue regeneration after *DE^ts^*>*hid*-induced tissue loss.

Next, we examined whether Spi/EGFR signaling is activated in the *DE^ts^*>*hid* model. *spi*-*lacZ* expression is induced in the ablated *GFP*-expressing dorsal domain of the disc compared to controls (Figure 7F,F′,G,G′). *kek-lacZ* as EGFR signaling marker is also strongly induced in the dorsal domain compared to controls (Figure 7H,H′,I,I′; arrow).

To determine if *spi* is genetically required in the *DE^ts^*>*hid* regeneration assay, we inactivated it by RNAi during the 30°C temperature shift, following the protocol in Figure 6E. In a control experiment, because *spi* is required for photoreceptor differentiation posterior to the morphogenetic furrow [79], [80], we tested if a 12 h *spi* RNAi treatment followed by 72 h recovery (R72 h) affects normal photoreceptor differentiation. However, eye discs treated in this way have a normal ELAV pattern (Figure 7C,C′; n = 20). After this control experiment, we tested for a genetic requirement of *spi* for regeneration of lost tissue due to *hid* expression. Strikingly, the regeneration response as judged by ELAV labeling was partially impaired when *spi* was inactivated by RNAi during *hid* induction (Figure 7D,D′; arrow). All experimental discs (n = 30) showed incomplete regeneration. The regeneration is only weakly affected, likely because *spi* is inactivated by RNAi only during the 30°C pulse during *hid* expression (Figure 6E) and is likely restored soon after reducing the temperature to 18°C. Nevertheless, in a heterozygous *spi* condition, the discs also incompletely regenerated after *DE^ts^*>*hid* treatment (Figure 7E,E′; N = 20). In summary, because *spi* RNAi and *spi* heterozygosity cause a partial failure to regenerate, these data imply a requirement of *spi* for regeneration in the ‘genuine’ *DE^ts^*>*hid* AiP model. Furthermore, these data support the notion that genetic screening using the simpler ‘undead’ AiP model can lead to identification of genes that may also have important roles for regeneration in ‘genuine’ AiP.

## Discussion

Apoptosis-induced proliferation (AiP) appears to be a mechanism by which developing organisms replace dying cells under stress conditions and initiate regenerative responses (reviewed by [37], [45]). In this paper, we described two AiP models in the developing *Drosophila* eye. The ‘undead’ *ey*>*hid*-*p35* model generates a hyperplastic overgrowth phenotype. To date this is the only known phenotype that provides a robust and convenient assay for genetic screening and identification of novel regulators of AiP. In contrast, we have not identified a similar robust and convenient phenotype that would allow direct screening for genes involved in ‘genuine’ (p35-independent) AiP and regeneration. Nevertheless, we developed the *DE^ts^*>*hid* model to verify genes identified in the ‘undead’ screen as being involved in ‘genuine’ AiP and regeneration.

Although the use of *p35* to keep dying cells in an ‘undead’ condition may be considered as unphysiological and artificial, to date all genes identified under *p35*-expressing conditions such as JNK, Wg and Spi, were also found to be involved in AiP in *p35*-independent models [42], [43] (this study). Furthermore, cancer cells may resemble ‘undead’ cells. They often initiate, but cannot execute the apoptotic program due to genetic loss or inactivation of effector caspases or other apoptotic components [81]–[84]. Such ‘undead’ cancer cells may contribute to tumor growth. Therefore, our *p35*-expressing AiP model could provide insights into new regulators of AiP as well as how impaired apoptosis may promote tumor growth.

Apoptotic caspases play a critical role for AiP. In *Drosophila*, the initiator caspase Dronc is required for activation of JNK activity which triggers AiP. However, it is unknown how Dronc activates JNK for AiP. Using RNAi, a specific subset of components in the JNK pathway were identified as required for AiP. The most upstream genes in the JNK pathway are Rho1 and Traf2. Traf2 appears to be an integration point for Eiger- and AiP-induced JNK activation, the latter one being mediated through Rho1 (Figure 2G). However, it is unknown how Dronc triggers Rho1 activation. It is unlikely that Dronc proteolytically cleaves Rho1 for two reasons. First, Rho1 does not contain a putative Dronc cleavage site [40], [85]. Second, a proteolytic cleavage is likely to destroy Rho1; however, our genetic analysis implies that *Rho1* function is required for AiP (Figure 2, Suppl. Figure S5). Therefore, it remains unknown how Dronc triggers Rho1 and thus JNK activation.

Interestingly, extracellular signaling pathways (Eiger/Wengen and Pvf/PVR) known to activate JNK [52] did not score as suppressors of AiP, suggesting that Dronc may autonomously activate JNK activity. This is also consistent with our observation that JNK activity occurs largely in *hid*- and *p35*-expressing clones (Figure 3). Nevertheless, it is also possible that a third extracellular signal is generated by ‘undead’ cells in a Dronc-dependent manner that triggers JNK activity in an autocrine and/or paracrine manner. The observation that in both ‘undead’ and ‘genuine’ AiP models JNK activity is also detectable in neighboring surviving cells (Figure 3) may support such a mechanism. Further work is necessary to reveal the exact mode of JNK activation by ‘undead’/dying cells.

In the ‘genuine’ (p35-independent) AiP model (*ptc^ts^*>*hid*), JNK activity is detectable in both dying and surviving cells. However, the surviving cells with increased JNK activity are also present in the *ptc* domain (Figure 3E,F) which was exposed to *hid* expression during the temperature shift. JNK activity is also restricted to the death domain in the *DE^ts^*>*hid* model (Suppl. Figure S7). Therefore, it is unclear whether a signaling mechanism from dying cells induces JNK activity in surviving cells, or whether the previous *hid* induction accounts for the JNK activity in surviving cells. It is also unclear how these cells survive. Even after a 16 h pulse of *hid* induction causing a strong apoptotic response in a large fraction of cells in the *ptc* domain, some cells survive (Figure 3F). They may receive survival signals from cells outside of the *ptc* domain, but that needs to be determined. These are interesting questions to be addressed in the future.

We have tested signaling pathways known to be involved in growth control for modification of the *ey>hid-p35* model. One example is the Hippo/Warts/Yorkie pathway [86], [87]. However, neither mutants of this pathway nor transcriptional reporters (*ex*-*lacZ*) scored positive in the *ey>hid-p35* model (data not shown). Therefore, at least in the eye disc, not every pathway involved in growth control is also involved in AiP. These observations stressed the necessity to perform unbiased genetic screens aimed at identifying the genes and mechanisms involved in AiP.

Therefore, we performed a pilot screen for modifiers of the *ey>hid-p35* AiP model using deficiencies of chromosome arm 2L. We identified four deficiencies as suppressors and three as enhancers (Table 1). Identification of AiP enhancers implies that there is also negative regulation of AiP. In one case we identified *spi*, encoding the *Drosophila* EGF ortholog, as a suppressor of AiP suggesting an involvement of EGFR signaling for AiP. This is further confirmed by the strong transcriptional induction of *spi* and an EGFR target gene, *kekkon*, in our AiP model. We also found that EGFR signaling is involved in an ‘undead’ AiP model in the wing and – more importantly – in the ‘genuine’ *DE^ts^*>*hid* regeneration model in the eye. The latter finding is crucial as it demonstrates that genes identified in the ‘undead’ screen may be relevant players for ‘genuine’ regeneration in response to apoptotic tissue loss. An involvement of EGFR and MAPK for regeneration is not unprecedented. It was previously shown that EGF is one of a few signals that stimulate hepatocyte proliferation during liver regeneration in mammals [88], [89]. In the Hydra regeneration model, apoptosis-induced proliferation depends on MAPK activation [90]. Therefore, these findings and considerations validate our screening approach using the ‘undead’ AiP model.

Identification of Spi/EGFR signaling as suppressor of AiP was unexpected because EGFR signaling negatively regulates the apoptotic activity of *hid* [91], [92]. Thus, by reducing EGFR activity, *hid* has increased apoptotic activity which is expected to induce even more AiP. Therefore, the AiP phenotype should be enhanced by heterozygosity of EGFR pathway components. However, the identification of *spi*, *Egfr*, *Dras* and *rl* as suppressor of AiP suggests that EGFR signaling is also required for AiP. One possibility to explain these two opposing functions of EGFR (negative regulation of *hid* and positive requirement for AiP) may be the exclusive appearance of Cas3*-positive areas and areas with EGFR activity (Figure 5F). Accordingly, while the Spi signal is generated in Cas3*-positive, apoptotic areas, it signals to neighboring Cas3*-negative, surviving areas to inactivate Hid and promote proliferation.

The identification of Spi/EGFR signaling may help to resolve a controversy about the signaling pathways involved in AiP. The exact roles of Wg and Dpp for AiP are unclear [26], [44], [46] and signaling by the EGFR pathway may contribute to the proliferative response in AiP.

Recently, a genetic screen has been reported aimed at identification and characterization of genes required for compensatory growth [93]. These authors induced apoptosis conditionally using a temperature-sensitive cell lethal mutant (*sec5^ts^*). Under normal conditions, the ablated tissue is replaced by new tissue due to compensatory proliferation. The authors then screened for mutants that fail to renew the lost tissue [93]. This was done in a clonal screen for chromosome arm 2L, the same chromosome arm we screened in the deficiency screen in our model. However, the genes identified in the *sec5^ts^* screen [93] do not map to the deficiencies that we have identified in our analysis (Table 1). Because it is unknown if *sec5^ts^*–induced compensatory proliferation requires caspase activity in apoptotic cells [93], it is not clear if this is a model of apoptosis-induced proliferation.

In summary, we have developed and tested the feasibility of the *ey>hid-p35* model for genetic screening. We are confident that this model will close gaps in our understanding of AiP regulation under *p35*-expressing conditions and in *p35*-independent regeneration. Finally, it will have implications for the understanding of tumorigenesis by ‘undead’ as well as ‘genuine’ apoptotic tumor cells [94].

## Materials and Methods

### Fly stocks and genetics

The following mutants and transgenic stocks were used: *dronc^I29^*; *ark^G8^*; *spi^1^*; *spi^01068^*; *Egfr^f2^*; *ras^ΔC40b^*, *rl^10a^*, *ey-Gal4*; *ptc*-*Gal4*; *DE*-*Gal4*; *tub*-*Gal80^ts^*; *UAS-p35*; *UAS-hid*; *UAS*-*GFP*; *wg-lacZ*; *puc-lacZ*; *kek1*-*lacZ*; *spi*-*lacZ* = *spi^01068^*; *TRE-dsRed*; *GMR*-*hid*; *GMR*-*Gal4 UAS*-*egr*. UAS-based RNAi stocks of the following genes were obtained from various stock centers (VDRC, Bloomington and NIG) and were tested for suppression of AiP: *dronc*, *dcp*-*1*, *drICE*; *bsk*, *egr*, *wgn*, *PVR*, *dTraf1*, *dTraf2*, *Rho1*, *msn*, *slpr*, *Tak1*, *dMekk1*, *dAsk1*, *hep*, *dMkk4*, *Jra*, *kay*, *spi*, *Egfr*. The exact genotype of *ey>hid-p35* is *UAS*-*hid*; *ey*-*Gal4 UAS*-*p35*/*CyO*,*tub*-*Gal80*. Expression of *tub*-*Gal80* in this stock suppresses the semi-lethality associated with *ey*-induced expression of *hid* and *p35*.

### Mosaic analysis

Larvae of the following genotype were heat shocked for 15min at 37°C, raised at room temperature for 48 h before they were analyzed at the late 3^rd^ instar larval stage. Genotype: *hs-FLP/UAS-hid; UAS-p35/act>y+>Gal4 UAS-GFP; puc-lacZ/+*.

### Tissue ablation using *ptc^ts^*>*hid* and *DE^ts^*>*hid*

Larvae of genotype *UAS-hid/+*; *ptc-Gal4 tub-Gal80^ts^/+*; *UAS-GFP/+* (Figure 3) and *UAS-hid/+*; *UAS-GFP/+*; *DE-Gal4 tub-Gal80^ts^/+* (Figure 6) were raised at 18°C. *hid* expression was induced by temporal temperature shift to 30°C for the indicated amount of time (Figure 3) or for 12 hours (Figure 6E). After the indicated recovery periods at 18°C, discs were dissected and analyzed as indicated in the panels.

### PH3 labelling and statistics in *DE^ts^*>*hid* model

Two rounds of experiments (ts12hR24h, at least 20 discs were analyzed each round) were done for both *DE^ts^>GFP* (control) and *DE^ts^>hid*. Increase of PH3-positive cells in dorsal eye disc portions of *DE^ts^>hid* animals are consistently observed. PH3-positive cell numbers were counted in dorsal (*GFP* ^+^) and ventral eye disc portions in selected discs. Size of the dorsal (*GFP* ^+^) and ventral eye disc portions were measured through the “histogram” function in Adobe Photoshop CS. To compare the density of PH3^+^ cells in each disc portion, number of PH3^+^ cells were divided by size (in pixels) of the corresponding tissue which is used to calculate the number of cells in 100,000 pixels (density). Such normalized density of PH3^+^ cells in various portions of eye discs (mean ± SD) were used for the statistical chart. PH3^+^ cell numbers in 100,000 pixels is on average 88 in *DE^ts^>hid* dorsal eye discs compared to 62 in the control dorsal discs. Their statistical significance was evaluated through a two-tailed, unpaired Student's t-Tests (P<0.04). In contrast, the number of PH3^+^ cells are comparable in ventral disc portions of each genotype suggesting that increased proliferation mostly occurred in the dorsal part of the disc (at least at the time point of R24 h).

### Immunohistochemistry

Imaginal discs were dissected from late 3^rd^ instar larvae and stained using standard protocols. Antibodies to the following primary antigens were used: PH3 (Upstate), anti-cleaved Caspase-3 (Cell Signaling), β-GAL (Promega), ELAV and Wg (DHSB). Secondary antibodies were donkey Fab fragments from Jackson ImmunoResearch. Images were taken with either a Zeiss AxioImager or a confocal microscope.

## Supporting Information

Figure S1 The JNK activity marker *TRE-dsRed* is induced in ‘undead’ *ey>hid-p35* cells. Shown are (A) wild-type (wt), (B) *ey>p35* and (C) *ey>hid-p35* eye imaginal discs labeled for dsRed (JNK marker, red in A–C; grey in A′–C′) and ELAV (photoreceptor neurons, green in A–C; grey in A″–C″). Only *ey>hid-p35* discs induce *TRE-dsRed* expression (C, C′; arrow) and disrupt the ELAV pattern (C″).
(TIF)

Figure S2 The *UAS-dcp-1^RNAi^* and *UAS-drICE^RNAi^* stocks are functional. Combined expression of *UAS-dcp-1^RNAi^* and *UAS-drICE^RNAi^* stocks suppresses both TUNEL-positive apoptosis (A,B) and eye-ablation of *GMR-hid* (C,D) suggesting that these stocks contain functional RNAi transgenes targeting *dcp-1* and *drICE*.
(TIF)

Figure S3 Several UAS-RNAi transgenes of the JNK pathway suppress *GMR-egr*. (A) The unmodified *GMR-Gal4 UAS-eiger* (*GMR>egr*) eye ablation phenotype. (B–H) RNAi transgenes targeting the genes indicated above the panels suppress the eye ablation phenotype induced by *GMR-Gal4 UAS-eiger* (*GMR>egr*) suggesting that they are functional.
(TIF)

Figure S4 Inactivation of JNK pathway genes in *ey*>*hid*-*p35* eye discs does not affect caspase activity. (A) A *ey*>*hid*-*p35* disc labeled for Cas3* and ELAV. (B) *dronc* RNAi suppresses Cas3* and normalizes the ELAV pattern in *ey*>*hid*-*p35* discs. (C–H) RNAi transgenes targeting the indicated JNK pathway components normalize the ELAV pattern, but fail to suppress Cas3* activity in *ey*>*hid*-*p35* discs suggesting that they suppress AiP downstream of caspase activation.
(TIF)

Figure S5 Rho1 acts upstream of JNK in the ‘undead’ AiP model. (A,A′,C,C′) *ey*>*hid*-*p35* discs are characterized by strong *puc-lacZ* (A,A′) and *wg* (C,C′) expression as well as disrupted photoreceptor pattern (ELAV). (B,B′,D,D′) RNAi targeting *Rho1* suppresses *puc*-*lacZ* (B,B′) and *wg* (D,D′) expression as well as normalizes the ELAV pattern in *ey*>*hid*-*p35* discs. Caspase activity is not affected suggesting that Rho1 acts downstream of Dronc and upstream of JNK.
(TIF)

Figure S6 *Egfr* is required for AiP in a wing model. (A) A control wing disc expressing *UAS*-*p35* under *nubbin* (*nub*)-*Gal4* (*nub*>*p35*) control shows normal Wg expression (A′) and little to no Cas3* labeling (A″). (B) An experimental AiP disc expressing *hid* and *p35* under *nub* control (*nub*>*hid*-*p35*) displays strong overgrowth with abnormal Wg pattern (B′) and strong Cas3* labeling (B″). Together with (D), these data suggests that *nub*>*hid*-*p35* is a suitable ‘undead’ AiP model. (C) A *nub*-*Gal4 UAS*-*p35* (*nub>p35*) control disc. *puc*-*lacZ* expression is detectable at low level. (D) Coexpression of *hid* and *p35* induces strong JNK activity (*puc*-*lacZ*) in the enlarged *nub* domain. (E) RNAi targeting *Egfr* suppresses the overrepresentation of the *nub* domain, but leaves *puc*-*lacZ* intact. This result suggests that EGFR signaling is required for AiP in the wing disc and acts downstream of JNK. (F,G) Control disc expressing *Egfr* RNAi in the *nub* domain without *hid*, in the presence (F) or absence (G) of *p35*. The size of the *nub* domain is not significantly altered by *Egfr* RNAi compared to (C).
(TIF)

Figure S7 Induction of the JNK activity marker *TRE-dsRed* in *DE^ts^*>*hid* eye imaginal discs. (A–D) *hid* and *GFP* expression were temporally induced for 12 h by temperature shift to 30°C during early third instar larval stage as indicated in Figure 6E. *dsRed* expression (red in A–D; grey in A′–D′; see arrows) was monitored at 0 h (A), 6 h (B), 12 h (C) and 24 h (D) recovery after the temperature shift. GFP (green in A–D; grey in A″–D″) marks the *DE* domain. Blue is DAPI labeling to outline the discs. *dsRed* labeling is weakly detectable at R0 h, peaks at 6 h after recovery and fades off at R12 h. At R24 h, it is barely visible. (E) A *DE^ts^*>*GFP* control disc at 6 h recovery after the temperature shift, labeled for dsRed (red in E, grey in E′). JNK activity is not induced. GFP expression in (E″) is strong. Blue in (E) is DAPI labeling to outline the discs.
(TIF)

Table S1 Chromosomal deficiencies tested in the AiP screen on 2L. Listed are the names of the deficiencies, the extent of the chromosomal deletions and the score in the AiP screen. Green marks suppressors and yellow marks enhancers. Deficiencies marked with * could not be scored, because they caused lethality in the *ey>hid-p35* background.
(PDF)
