# Supplementary figures and images for: Genetic Models of Apoptosis-Induced Proliferation Decipher Activation of JNK and Identify a Requirement of EGFR Signaling for Tissue Regenerative Responses in Drosophila
Source: PLoS Genet. 2014 Jan 30;10(1):e1004131. doi: 10.1371/journal.pgen.1004131 (PMC3907308; doi:10.1371/journal.pgen.1004131)

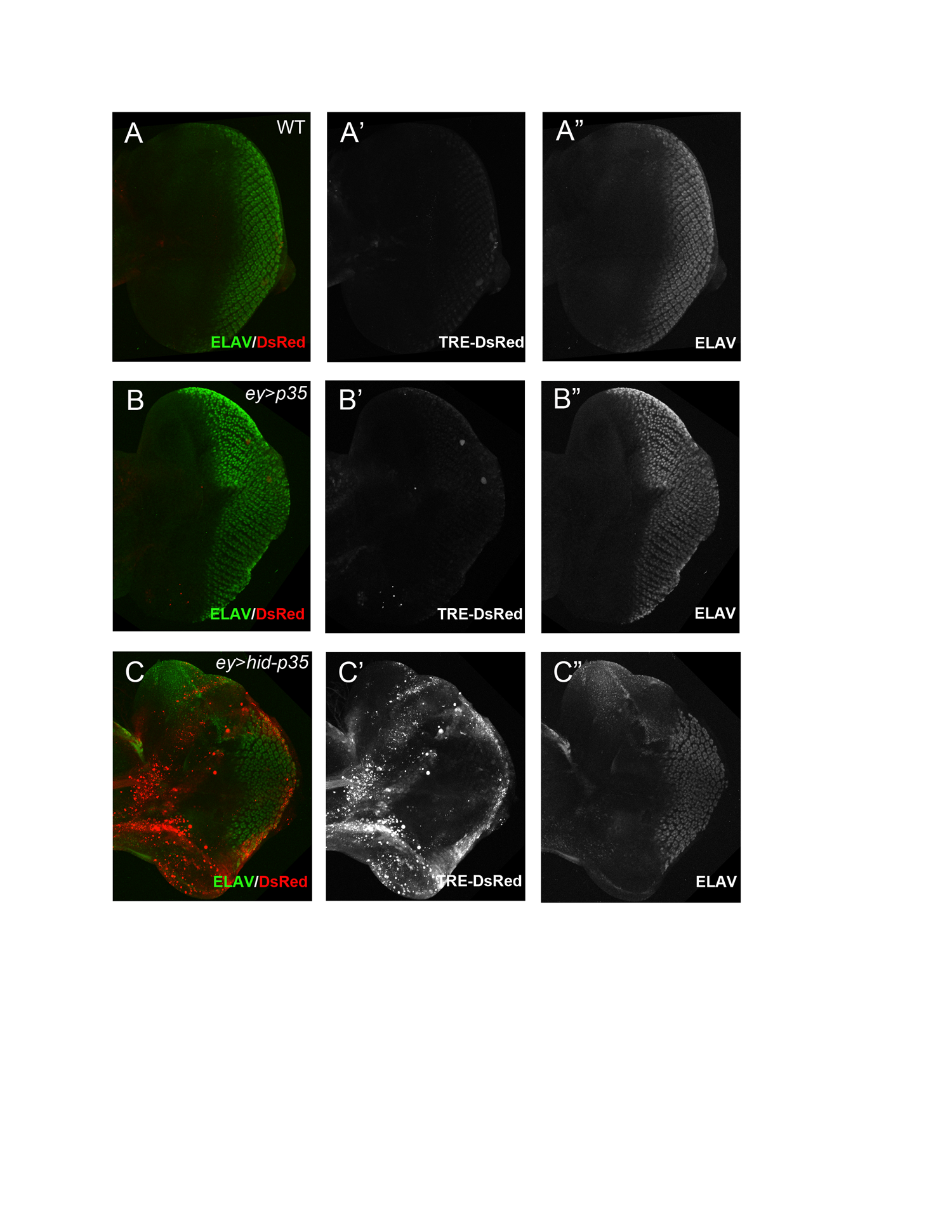

Supplement: Figure S1 — The JNK activity marker TRE-dsRed is induced in ‘undead’ ey>hid-p35 cells. Shown are (A) wild-type (wt), (B) ey>p35 and (C) ey>hid-p35 eye imaginal discs labeled for dsRed (JNK marker, red in A–C; grey in A′–C′) and ELAV (photoreceptor neurons, green in A–C; grey in A″–C″). Only ey>hid-p35 discs induce TRE-dsRed expression (C, C′; arrow) and disrupt the ELAV pattern (C″). (TIF) [file pgen.1004131.s001.tif]

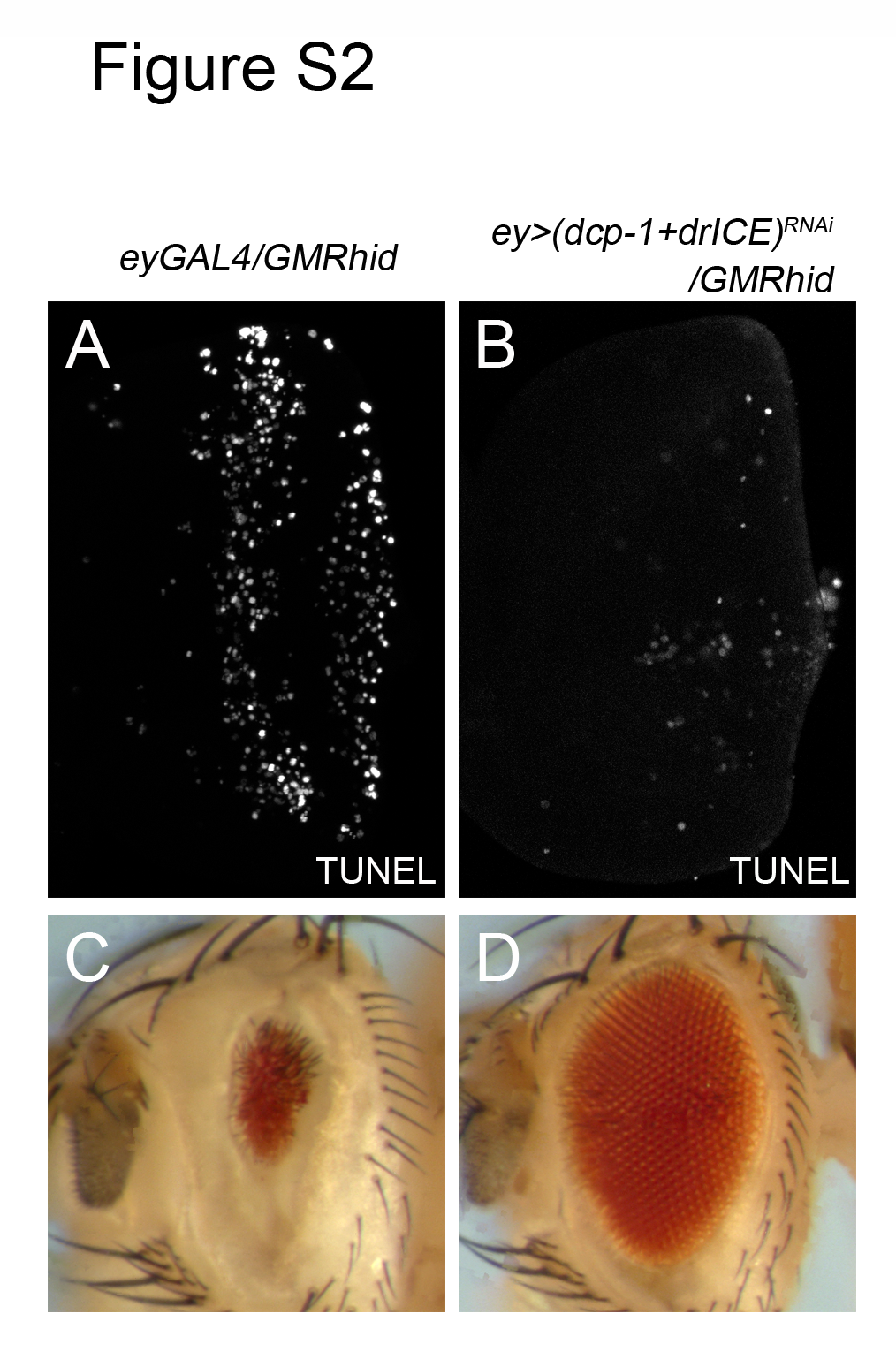

Supplement: Figure S2 — The UAS-dcp-1RNAi and UAS-drICERNAi stocks are functional. Combined expression of UAS-dcp-1RNAi and UAS-drICERNAi stocks suppresses both TUNEL-positive apoptosis (A,B) and eye-ablation of GMR-hid (C,D) suggesting that these stocks contain functional RNAi transgenes targeting dcp-1 and drICE. (TIF) [file pgen.1004131.s002.tif]

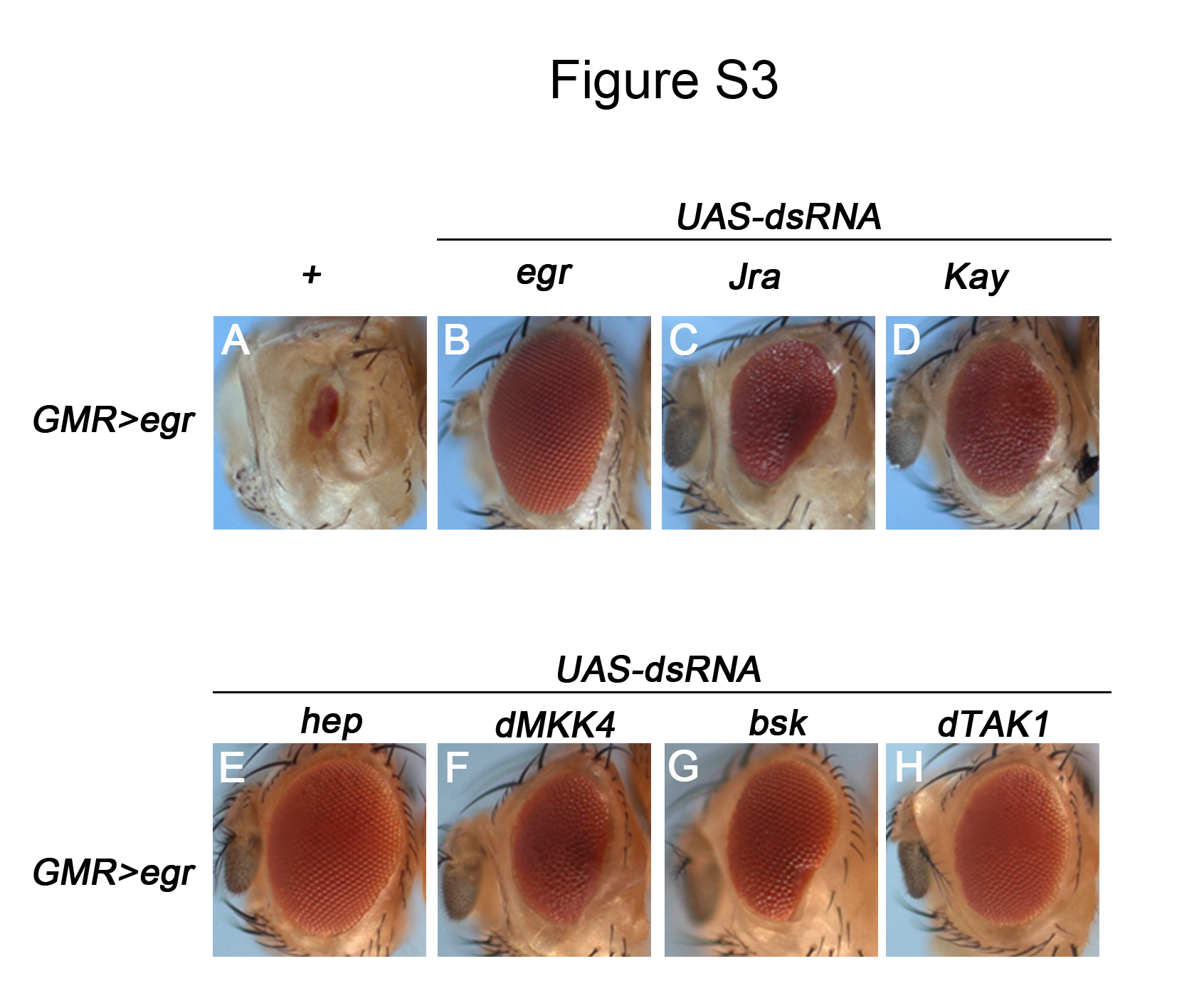

Supplement: Figure S3 — Several UAS-RNAi transgenes of the JNK pathway suppress GMR-egr. (A) The unmodified GMR-Gal4 UAS-eiger (GMR>egr) eye ablation phenotype. (B–H) RNAi transgenes targeting the genes indicated above the panels suppress the eye ablation phenotype induced by GMR-Gal4 UAS-eiger (GMR>egr) suggesting that they are functional. (TIF) [file pgen.1004131.s003.tif]

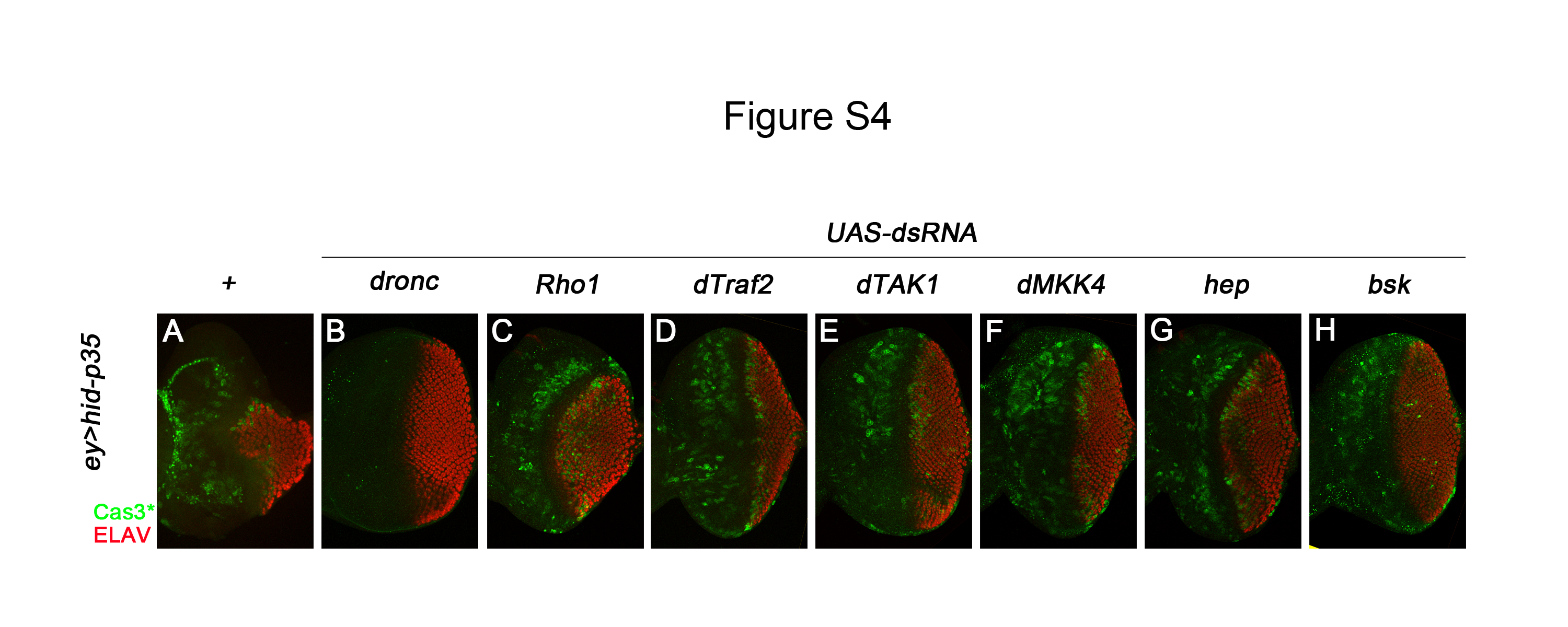

Supplement: Figure S4 — Inactivation of JNK pathway genes in ey>hid-p35 eye discs does not affect caspase activity. (A) A ey>hid-p35 disc labeled for Cas3* and ELAV. (B) dronc RNAi suppresses Cas3* and normalizes the ELAV pattern in ey>hid-p35 discs. (C–H) RNAi transgenes targeting the indicated JNK pathway components normalize the ELAV pattern, but fail to suppress Cas3* activity in ey>hid-p35 discs suggesting that they suppress AiP downstream of caspase activation. (TIF) [file pgen.1004131.s004.tif]

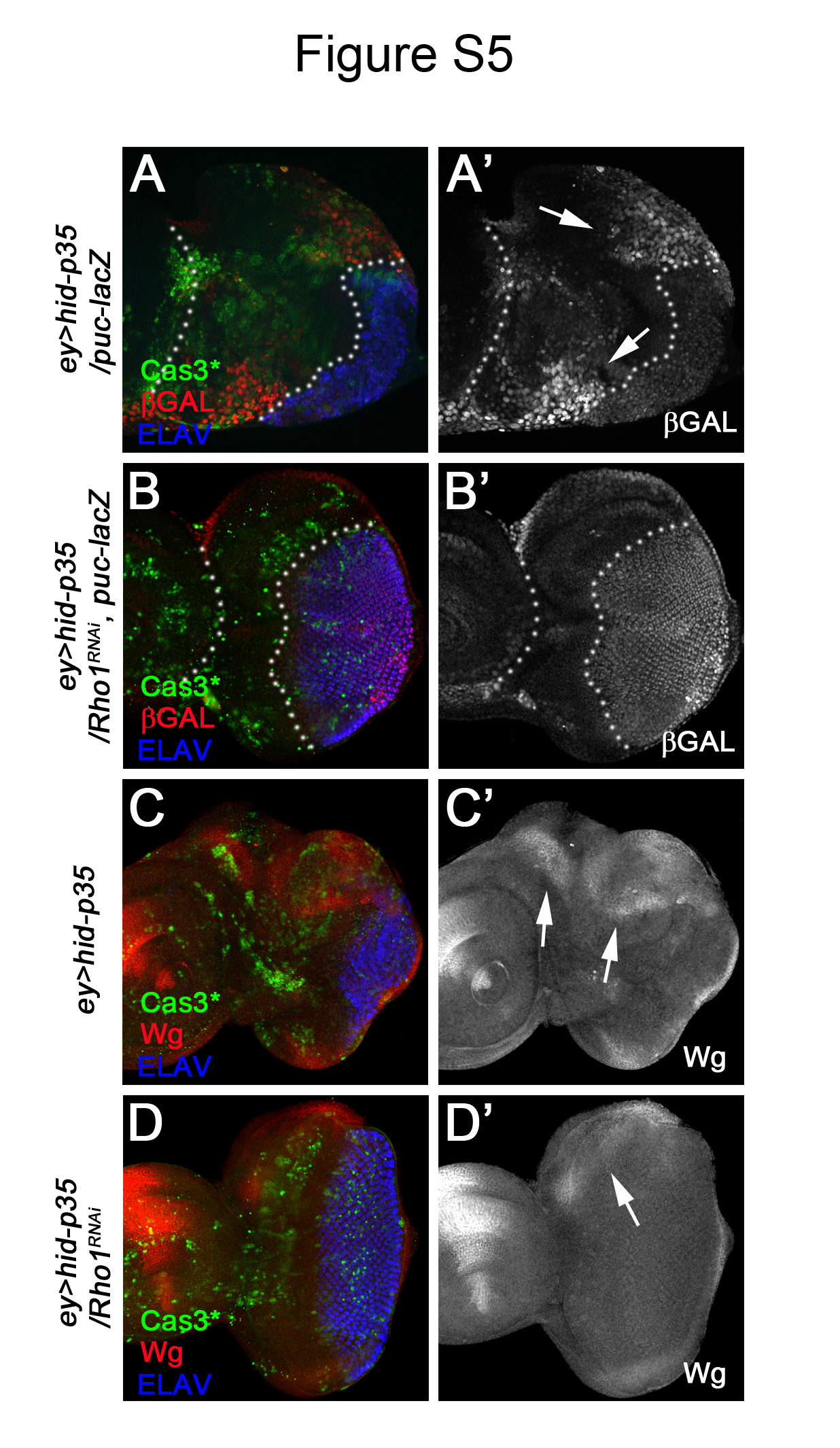

Supplement: Figure S5 — Rho1 acts upstream of JNK in the ‘undead’ AiP model. (A,A′,C,C′) ey>hid-p35 discs are characterized by strong puc-lacZ (A,A′) and wg (C,C′) expression as well as disrupted photoreceptor pattern (ELAV). (B,B′,D,D′) RNAi targeting Rho1 suppresses puc-lacZ (B,B′) and wg (D,D′) expression as well as normalizes the ELAV pattern in ey>hid-p35 discs. Caspase activity is not affected suggesting that Rho1 acts downstream of Dronc and upstream of JNK. (TIF) [file pgen.1004131.s005.tif]

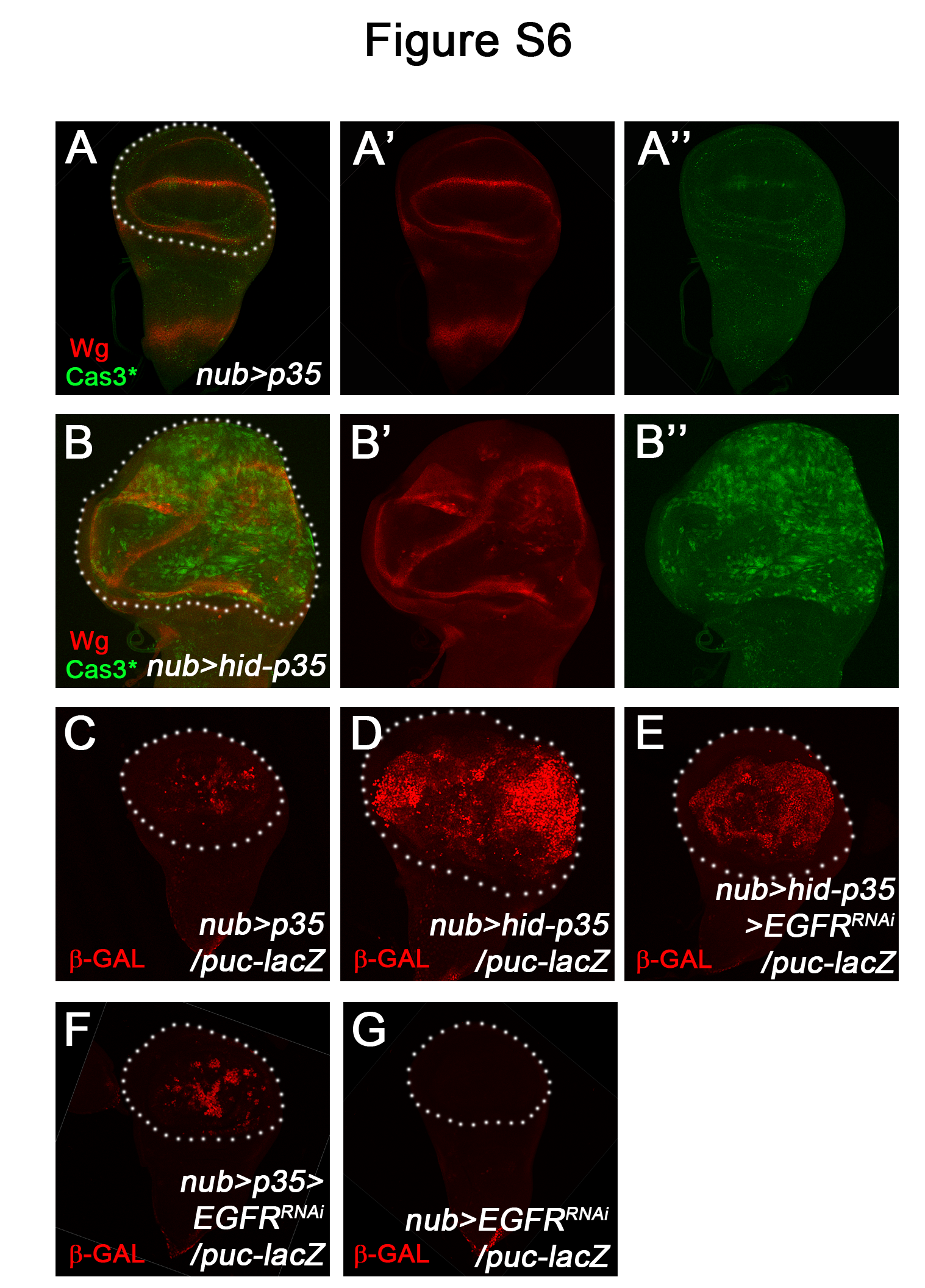

Supplement: Figure S6 — Egfr is required for AiP in a wing model. (A) A control wing disc expressing UAS-p35 under nubbin (nub)-Gal4 (nub>p35) control shows normal Wg expression (A′) and little to no Cas3* labeling (A″). (B) An experimental AiP disc expressing hid and p35 under nub control (nub>hid-p35) displays strong overgrowth with abnormal Wg pattern (B′) and strong Cas3* labeling (B″). Together with (D), these data suggests that nub>hid-p35 is a suitable ‘undead’ AiP model. (C) A nub-Gal4 UAS-p35 (nub>p35) control disc. puc-lacZ expression is detectable at low level. (D) Coexpression of hid and p35 induces strong JNK activity (puc-lacZ) in the enlarged nub domain. (E) RNAi targeting Egfr suppresses the overrepresentation of the nub domain, but leaves puc-lacZ intact. This result suggests that EGFR signaling is required for AiP in the wing disc and acts downstream of JNK. (F,G) Control disc expressing Egfr RNAi in the nub domain without hid, in the presence (F) or absence (G) of p35. The size of the nub domain is not significantly altered by Egfr RNAi compared to (C). (TIF) [file pgen.1004131.s006.tif]

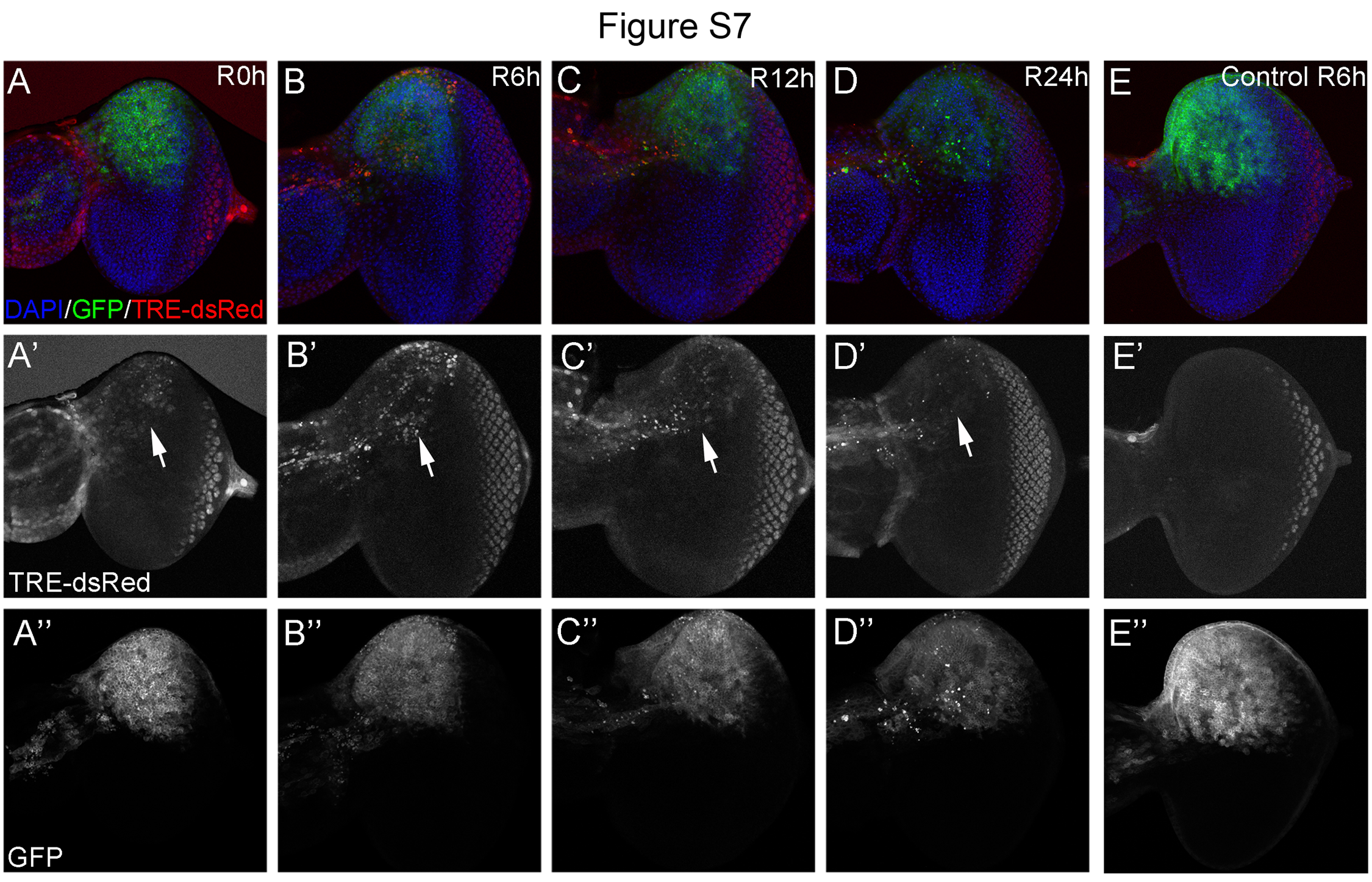

Supplement: Figure S7 — Induction of the JNK activity marker TRE-dsRed in DEts>hid eye imaginal discs. (A–D) hid and GFP expression were temporally induced for 12 h by temperature shift to 30°C during early third instar larval stage as indicated in Figure 6E. dsRed expression (red in A–D; grey in A′–D′; see arrows) was monitored at 0 h (A), 6 h (B), 12 h (C) and 24 h (D) recovery after the temperature shift. GFP (green in A–D; grey in A″–D″) marks the DE domain. Blue is DAPI labeling to outline the discs. dsRed labeling is weakly detectable at R0 h, peaks at 6 h after recovery and fades off at R12 h. At R24 h, it is barely visible. (E) A DEts>GFP control disc at 6 h recovery after the temperature shift, labeled for dsRed (red in E, grey in E′). JNK activity is not induced. GFP expression in (E″) is strong. Blue in (E) is DAPI labeling to outline the discs. (TIF) [file pgen.1004131.s007.tif]
