# Supplementary material for: Genetic Models of Apoptosis-Induced Proliferation Decipher Activation of JNK and Identify a Requirement of EGFR Signaling for Tissue Regenerative Responses in Drosophila
Source: PLoS Genet. 2014 Jan 30;10(1):e1004131. doi: 10.1371/journal.pgen.1004131 (PMC3907308; doi:10.1371/journal.pgen.1004131)
Supplement: Table S1 — Chromosomal deficiencies tested in the AiP screen on 2L. Listed are the names of the deficiencies, the extent of the chromosomal deletions and the score in the AiP screen. Green marks suppressors and yellow marks enhancers. Deficiencies marked with * could not be scored, because they caused lethality in the ey>hid-p35 background. (PDF) [file pgen.1004131.s008.pdf]

Supplemental Table S1. Chromosomal deficiencies tested in the AiP screen on 2L.

* These deficiencies could not be scored because of lethality of ey>hid-p35/Df flies.

Deficiencies	Deleted Segments	Effects on ey>hid-p35
Df(2L)net-PMC	21A1;21B6-7	no effect
Df(2L)ED19	21B3;21B7	no effect
Df(2L)BSC106	21B7;21C2	no effect
Df(2L)BSC4	21B7-C1;21C2-3	no effect
Df(2L)BSC16	21C3-4;21C6-8	no effect
Df(2L)S2	21C6-D1; 22A6-B1	no effect
Df(2L)ast2	21D1-21D2;22B2-22B3	no effect
Df(2L)frtz17	21E3-4;22B5-7	no effect
Df(2L)Exel6006	22B5;22D1	no effect
Df(2L)ED123	22B8;22D4	enhancement
Df(2L)BSC37	22D2-3;22F1-2	no effect
Df(2L)dpp[d14]	22E4-F2;22F3-23A1	no effect
Df(2L)C144	22F4-23A1;23C2-4	suppression
Df(2L)ED136	22F4;23A3	suppression
Df(2L)Exel6277	23A2;23B1	no effect
Dp(2;1)JS13	23A3;23D6	no effect
Df(2L)ED206	23B6;23D1	suppression
Df(2L)JS17	23C1-2;23E1-2	suppression
Df(2L)JS32	23C3;23D2	no effect
Df(2L)Exel7014	23C4;23C5	suppression
Df(2L)BSC162	23C5;23D4	no effect
Df(2L)BSC28	23C5-D1;23E2	suppression
Df(2L)S2590	23D2;23E3	no effect
Df(2L)Exel8008	23E3;23E5	no effect
Df(2L)BSC31	23E5;23F4-5	suppression
Df(2L)tim-02	23F2-3;23F6-24A1	no effect
Df(2L)Exel7016	23F3;23F3	no effect
Df(2L)drm-P1	23F3-4;24A1	no effect
Df(2L)BSC292	23F6;24A2	no effect
Df(2L)ed1	24A2;24D4	no effect
Df(2L)sc19-7	24D2-4;25C2-3	no effect
Df(2L)Exel8012	25B1;25B5	no effect
Df(2L)Exel7022	25B10;25C3	no effect
Df(2L)Exel7021	25B3;25B9	no effect
Df(2L)Exel8013	25B5;25B10	no effect
Df(2L)BSC110	25C1;25C4	no effect
Df(2L)BSC109	25C4;25C8	no effect
Df(2L)Exel6011	25C8;25D5	lethality*
Df(2L)cl-h3	25D2-4;26B2-5	no effect

Df(2L)BSC5	26B1-2;26D1-2	no effect
Df(2L)ED384	26B2;26D7	no effect
Df(2L)BSC7	26D10-E1;27C1	no effect
Df(2L)BSC6	26D3-E1;26F4-7	enhancement
Df(2L)BSC9	26F5-7;27A2-B2	no effect
Df(2L)ED6569	27A1;27C4	no effect
Df(2L)J-H	27C2-9;28B3-4	no effect
Df(2L)ade3	27D1-2;27F1-2	no effect
Df(2L)BSC41	28A4-B1;28D3-9	no effect
Df(2L)Exel6018	28B1;28C1	no effect
Df(2L)ED508	28B1;28C4	enhancement
Df(2L)Trf-C6R31	28DE;28DE	no effect
Df(2L)Exel7034	28E1;28F1	lethality*
Df(2L)TE29Aa-11	28E4-7;29B2-C1	no effect
Df(2L)ED611	29B4;29C3	enhancement
Df(2L)N22-5	29C3;30C9	no effect
Df(2L)gamma7	30A9-B1;30D2-F4	no effect
Df(2L)BSC17	30C3-5;30F1	no effect
Df(2L)Exel7043	30D1;30F1	no effect
Df(2L)Mdh	30D-30F;31F	no effect
Df(2L)BSC205	30F5;31A2	no effect
Df(2L)Exel9032	31A3;31B1	no effect
Df(2L)J1	31B;31D	no effect
Df(2L)BSC206	31B1;31D9	no effect
Df(2L)J3	31D;31F	no effect
Df(2L)Exel7048	31E3;31F5	enhancement
Df(2L)ED746	31F4;32A5	no effect
Df(2L)BSC32	32A1-2;32C5-D1	no effect
Df(2L)FCK-20	32D1;32F1-3	no effect
Df(2L)Prl	32F1-3;33F1-2	no effect
Df(2L)prd1.7	33B3;34A1-34A2	no effect
Df(2L)ED778	33E9;34A7	no effect
Df(2L)BSC30	34A3;34B7-9	no effect
In(2L)b82a1	34B7;34E3	no effect
Df(2L)BSC147	34C1;34C6	no effect
Df(2L)b80e3	34C4;35A4	no effect
Df(2L)64j	34D1;35C1	no effect
Df(2L)ED1050	35B8;35D4	enhancement
Df(2L)Sco[rv14]	35D1;35E2	no effect
Df(2L)RA5	35E1;36A1	no effect
Df(2L)Exel7066	36A1;36A12	no effect
Df(2L)Exel7067	36A12;36B2	no effect
Df(2L)Exel8036	36B1;36C9	no effect
Df(2L)TW137	36C2-4;37B9-C1	suppression

Df(2L)TW119	36C4;36E1	no effect
Df(2L)M36F-S5	36D1;36F11	no effect
Df(2L)TW50	36E4;38A7	no effect
Df(2L)M36F-S6	36E6-F1;36F7-9	no effect
Df(2L)Exel6041	36F6;37A2	no effect
Df(2L)Exel7071	37A1;37A4	no effect
Df(2L)Exel7072	37A2;37B6	no effect
Df(2L)BSC341	37B11;37D3	no effect
Df(2L)hk-UC1	37B2;37B13	no effect
Df(2L)Exel6042	37B8;37C5	no effect
Df(2L)ED1226	37B9;37E3	no effect
Df(2L)Exel8040	37C1;37C5	no effect
Df(2L)Exel6043	37C5;37D7	no effect
Df(2L)ED1231	37C5;37E3	no effect
Df(2L)ED1272	37C5;38A2	suppression
Df(2L)Exel8041	37D7;37F2	no effect
Df(2L)ED1303	37E5;38C6	suppression
Df(2L)ED1317	38D1;38F5	no effect
Df(2L)Exel7080	38F3;39A2	enhancement
Df(2L)DS6	38F5;39E7-F1	no effect
Df(2L)Exel6047	39A2;39B4	no effect
Df(2L)ED1473	39B4;40A5	no effect
Df(2L)BSC151	40A5;40E5	no effect
